# Thiol-Ene Photo-Click Hydrogels with Tunable Mechanical Properties Resulting from the Exposure of Different -Ene Moieties through a Green Chemistry

**DOI:** 10.3390/ma16052024

**Published:** 2023-02-28

**Authors:** Rossella Laurano, Monica Boffito, Claudio Cassino, Ludovica Midei, Roberta Pappalardo, Valeria Chiono, Gianluca Ciardelli

**Affiliations:** 1 Department of Mechanical and Aerospace Engineering, Politecnico di Torino, 10129 Turin, Italy; 2 Department of Science and Technological Innovation, Università del Piemonte Orientale, 15121 Alessandria, Italy; 3 Department of Surgical Sciences, Università degli Studi di Torino, 10126 Turin, Italy

**Keywords:** green functionalization, biomaterial design, thiol-ene formulations, Vis-light irradiation, dual stimuli-responsive hydrogels

## Abstract

Temperature and light responsiveness are widely exploited stimuli to tune the physico-chemical properties of double network hydrogels. In this work, new amphiphilic poly(ether urethane)s bearing photo-sensitive moieties (i.e., thiol, acrylate and norbornene functionalities) were engineered by exploiting the versatility of poly(urethane) chemistry and carbodiimide-mediated green functionalization procedures. Polymers were synthesized according to optimized protocols maximizing photo-sensitive group grafting while preserving their functionality (approx. 1.0 × 10^19^, 2.6 × 10^19^ and 8.1 × 10^17^ thiol, acrylate and norbornene groups/g_polymer_), and exploited to prepare thermo- and Vis-light-responsive thiol-ene photo-click hydrogels (18% *w*/*v*, 1:1 thiol:ene molar ratio). Green light-induced photo-curing allowed the achievement of a much more developed gel state with improved resistance to deformation (*ca.* 60% increase in critical deformation, γL). Triethanolamine addition as co-initiator to thiol-acrylate hydrogels improved the photo-click reaction (i.e., achievement of a better-developed gel state). Differently, L-tyrosine addition to thiol-norbornene solutions slightly hindered cross-linking, resulting in less developed gels with worse mechanical performances (~62% γL decrease). In their optimized composition, thiol-norbornene formulations resulted in prevalent elastic behavior at lower frequency compared to thiol-acrylate gels due to the formation of purely bio-orthogonal instead of heterogeneous gel networks. Our findings highlight that exploiting the same thiol-ene photo-click chemistry, a fine tuning of the gel properties is possible by reacting specific functional groups.

## 1. Introduction

Hydrogels are three-dimensional (3D) networks of cross-linked polymeric chains capable to retain and absorb a huge amount of biological fluids and with a soft and rubbery consistency that makes them resemble human soft tissues [1]. Over the last decades, hydrogels have found widespread application in the biomedical field, as cell carriers [2,3,4], biomaterial inks or bioinks [5,6,7], fillers of 3D porous matrices based on thermoplastic polymers [8,9], and drug delivery systems [10,11,12].

Hydrogel formulations can be classified according to different parameters, such as their cross-linking method, stimuli-responsiveness, composition, configuration, source, preparation method and ionic charge [13,14]. Based on the adopted cross-linking method, hydrogels can be classified into physical or chemical formulations. In physical hydrogels, the sol-to-gel transition process is driven by the occurrence of physical, non-covalent interactions among the polymeric chains, such as ionic bonds, hydrophobic interactions, and hydrogen bonds [15]. Their gelation requires neither the addition of potential toxic crosslinking agents/organic solvents nor the irradiation with light. Moreover, their sol-to-gel transition does not release heat at the gelation site, that could induce denaturation of incorporated proteins (e.g., growth factors), degradation of encapsulated drugs or damages to embedded cells/surrounding tissues. Furthermore, physical hydrogel formulations are usually suitable for mini-invasive application thanks to their characteristic injectability through traditional needles and in situ gelation potential. However, physical hydrogels generally suffer for poor stability in watery environment that does not make them suitable for long-term applications (e.g., for prolonged drug delivery, as forming materials of 3D bioprinted structs). Moreover, they usually exhibit weak mechanical properties that result in a very soft consistency and incapability to keep the shape for prolonged time intervals or during transport or storage.

On the other hand, chemical hydrogels rely on covalent bonds that form among their constituent polymeric chains [15]. Chemical hydrogels can be prepared by free radical or condensation reactions, leading to the formation of a permanent chemically-cross-linked gel network. Such class of hydrogels overcomes many drawbacks of physical formulations [16]: they exhibit higher stability in aqueous environment and good mechanical strength due to the covalent crosslinks present in their network. As a result, they are much more suitable for long-term applications and when specific mechanical performances and shape retention and fidelity are required (e.g., in 3D bioprinted structs). Moreover, their gelation is usually highly controllable, enabling a fine tuning of the physico-chemical properties of the resulting gel network. However, the sol-to-gel transition of these formulations often requires the addition of chemical crosslinkers to the hydrogel or the application of high intensity UV light irradiation with consequent toxicity-related issues. Moreover, the use of organic solvents or UV light irradiation results in additional issues regarding the risk of denaturation/degradation of embedded proteins/drugs. Last but not least, the formation of strong networks upon UV light irradiation does not allow their mini-invasive application when used as delivery systems.

More recently, the pros of both physical and chemical hydrogels have been combined into double network formulations that rely on the concurrent presence of both physical and chemical interactions [17,18,19,20,21,22,23,24]. The approach provides hydrogel formulations with additional degrees of freedom as the tuning of their physico-chemical properties can be conducted working on both the physically- and the chemically-cross-linked counterparts. This enables a fine modulation of gelation kinetics, swelling properties, degradation/dissolution profiles and mechanical performances and expands the application fields of these systems. Indeed, such approach allows the engineering of hydrogel formulations able to completely fulfill the specific requisites of the envisaged application. For instance, in the case of cell embedding, the hydrogel can be ad-hoc engineered to in vitro recapitulate the mechanical properties of the targeted tissue/organ, thus enabling a better mimesis of the native environment and a proper stimulation of cell behavior (e.g., differentiation towards a specific cell phenotype in the case of stem cell encapsulation). This is also useful when hydrogels are used for the fabrication of 3D bioprinted structs that will be properly integrated in the host tissue at the implantation site. Similarly, 3D bioprinted constructs with optimal physico-chemical properties will finely replicate the native tissue/organ properties in the design of bioengineered in vitro models. Additionally, the double cross-linking process can be exploited to provide a gradually increasing stability to these formulations, enabling a first manipulation (e.g., injection or extrusion) in mild conditions coupled with a primary stability provided by physical cross-links, and a subsequent stabilization through the formation of permanent bonds among the polymeric chains. Lastly, for drug delivery applications the double cross-linked network can be optimized to achieve the desired payload release profile that maximizes therapeutic efficacy, while minimizing side effects and the need of repetitive administrations. In this regard, the selection of a proper crosslinking method represents a crucial point to: (i) ensure the integrity of encapsulated molecules, (ii) preserve hydrogel injectability for its mini-invasive administration, and (iii) achieve mechanical properties suitable to ensure hydrogel resistance against dissolution. A possible approach to design double network formulations lies in the combination of thermo- and light-responsiveness which provide physical and chemical gels, respectively. These features can be applied using the same polymer backbone, as in the case of methacryloyl gelatin [24] and Pluronic^®^ diacrylate [25,26], or can result from a blending procedure between thermo- and light-sensitive polymers, such as Pluronic/poly(ethylene glycol) diacrylate blends [27]. Irrespective of the adopted strategy, two critical issues can be mainly identified, namely the use of UV light which can potentially damage the encapsulated payload [28,29], and the loss of system injectability due to the achievement of too strong gel networks [30].

**Figure 1:**
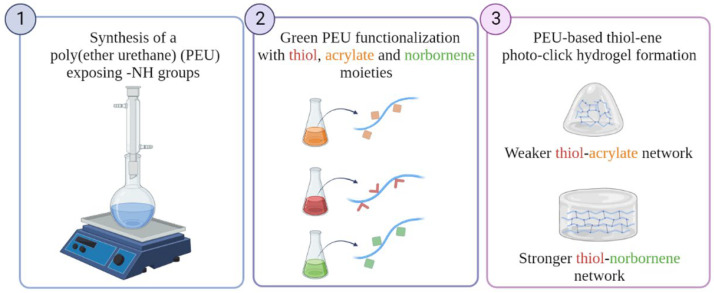
Schematic representation of the concept underpinning the work: (1) synthesis of an amphiphilic poly(ether urethane) bearing secondary amino groups; (2) polymer functionalization with different photo-sensitive moieties by exploiting the water-based carbodiimide chemistry, and (3) formulation of thiol-ene photo-click hydrogels characterized by a double network formed as a consequence of temperature increase followed by exposure to visible light, and a different strength based on the -ene moiety.

Moving from these premises, this work was aimed at the engineering of double network formulations based on thiol-ene photo-click chemistry with features able to overcome previously discussed criticisms. Specifically, this purpose was achieved by exploiting our group’s knowledge on poly(urethane) and carbodiimide chemistries [31,32,33,34,35] and by using light in the visible range as more payload-friendly irradiation source [36] (Figure 1). Specifically, a secondary amine-bearing thermo-sensitive amphiphilic Poloxamer^®^ 407-based poly(ether urethane) (PEU) was first synthesized and then, further subjected to bulk functionalization via the water-based eco-friendly carbodiimide-chemistry to expose light-responsive moieties. In detail, the -NH groups exposed along PEU polymeric chains were reacted with thioglycolic acid (TGA), 2-carboxyethyl acrylate (CEA) and 5-norbornene-2-carboxylic acid (NBE) to expose sulfhydryl, acrylate and norbornene groups, respectively. While TGA grafting has been recently optimized and reported by our group [34], the reaction conditions to maximize the grafting yield of CEA or NBE to the PEU backbone, while preserving the chemical structure of the exposed functional groups, have been optimized in the present work. Then, temperature- and light-sensitive hydrogels were designed by combining the TGA-functionalized PEU with CEA- or NBE-grafted PEU to obtain double network formulations in which chemical cross-linking occurred via thiol-ene chemistry. In order to induce the chemical cross-linking among the polymeric chains, we exploited Vis-light irradiation that is recently taking advantage over UV-light irradiation being a more cell- and eco-friendly light source and requiring more cytocompatible type II photo-initiators compared to type I [36]. Furthermore, Vis-light thiol-ene photo-click chemistry represents a powerful tool, ensuring high spatio-temporal control and bio-orthogonal networks [37], thus opening the way to finely tune construct mechanical properties and stability, being them very important aspects for their application as in vitro tissue models [38]. In this perspective, in this contribution, we thoroughly studied the influence of the –ene moiety (either acrylate or norbornene) on the photo-crosslinking mechanism and hydrogel mechanical properties by spectroscopic and rheological analyses. In parallel, we also investigated the contribution on the initiation efficiency potentially deriving from the addition of a co-initiator (i.e., triethanolamine (TEOA) [39] and L-Tyrosine (TYR) [40] for thiol-acrylate and thiol-norbornene systems, respectively) to the developed thiol-ene formulations. To the best of our knowledge this work reports for the first time the use of rheology as a tool to study irradiated hydrogel mechanical properties, taking advantage of the retained system capability to undergo temperature-driven gel-to-sol transition even after light exposure.

## 2. Materials and Methods

### 2.1. Materials

The triblock co-polymer Poloxamer^®^ 407 (P407, poly(ethylene oxide)-poly(propylene oxide)-poly(ethylene oxide), PEO-PPO-PEO, ${\bar{M}}_{n}$ 12,600 Da, 70% *w*/*w* PEO), the aliphatic diisocyanate 1,6-hexamethylene diisocyanate (HDI), the catalyzer dibutyltin dilaurate (DBTDL) and the chain extender N-Boc diethanolamine were purchased from Merck (Milan, Italy). Prior to the synthesis, P407 was dried under dynamic vacuum at 100 °C for 8 h and then, cooled down to 40 °C under static pressure condition (200 mbar) until use, HDI was distilled under reduced pressure, N-Boc diethanolamine was kept at room temperature (RT) under vacuum in a desiccator, and 1,2-dichloroethane (DCE) was anhydrified over activated molecular sieves (3Å, Merck, Milan, Italy) under nitrogen atmosphere overnight. All solvents were purchased from Carlo Erba Reagents (Milan, Italy) in analytical grade and used as received if not further specified.

### 2.2. Poly(ether urethane) Synthesis and -NH Group Exposure

The amphiphilic PEU used in this work was synthesized through a two-step procedure according to the protocol previously reported by Laurano et al. [34]. Briefly, in the first step of the synthesis, the macrodiol P407 was dissolved at 15% *w/v* in anhydrous DCE and reacted with HDI (2:1 molar ratio with respect to P407) at 80 °C for 45 min upon the addition of a catalytic amount of DBTDL (0.1% *w*/*w* with respect to P407). Then, the temperature was lowered to 60 °C and N-Boc diethanolamine (5% *w/v* in anhydrous DCE) was added to the mixture at 1:1 molar ratio with respect to the macrodiol to start the chain extension step. After 120 min, the system was equilibrated at RT and the reaction was stopped by adding anhydrous methanol. The polymer was collected through precipitation in petroleum ether (4:1 volume ratio with respect to DCE total volume) and dried under the fume-hood before purification. Lastly, the synthesized PEU was dissolved at 20% *w/v* in DCE, purified by precipitating the polymeric solution in a mixture of diethyl ether and methanol 98:2 *v/v* at 5:1 volume ratio with respect to the used DCE volume and collected by centrifugation (Hettich, MIKRO 220R, Tuttlingen, Germany) at 0 °C, 6000 rpm for 20 min. The obtained PEU was dried overnight under the fume-hood and then stored at 4 °C under vacuum atmosphere until use. This polymer will be referred to with the acronym DHP407, where D, H and P407 stand for the chain extender, diisocyanate and macrodiol, respectively.

Subsequently, Boc caging groups were removed by subjecting DHP407 to an acidic treatment in chloroform/trifluoroacetic acid (CF/TFA) 90/10 *v/v* [33] thus, resulting in the exposure of secondary amino groups along PEU chains. Specifically, the PEU was first dissolved in CF for 2 h, at RT, under magnetic stirring (250 rpm) and nitrogen atmosphere; then, TFA was added (4% *w/v* polymeric concentration, 90/10 *v/v* CF/TFA) and the reaction was carried on for an additional hour under the same conditions. Subsequently, the deprotected PEU (acronym D-DHP407) solution was concentrated in a rotary evaporator (Buchi Rotavapor Labortechnik AG, Flawil, Switzerland) and washed twice with 100 mL of CF to completely remove residual TFA traces. Lastly, D-DHP407 was dispersed in 200 mL of double demineralized water (ddH_2_O) at 4 °C overnight, dialyzed (10–12 kDa cut-off membrane, Merck, Milan, Italy) for 2 days to wash Boc groups out and freeze-dried using a Martin Christ ALPHA 2-4 LSC (Osterode am Harz, Germany).

### 2.3. Poly(ether urethane) Chemical Characterization and -NH Group Quantification

The success of the synthesis procedure and the absence of degradation phenomena induced by the cleavage of Boc protecting groups were assessed step-by-step through Attenuated Total Reflectance Fourier Transform Infrared (ATR-FTIR) spectroscopy and Size Exclusion Chromatography (SEC). Furthermore, exposed -NH groups were colorimetrically quantified through the Orange II Sodium Salt assay. All these characterizations were conducted according to previously published protocols [33].

### 2.4. Poly(ether urethane) Functionalization with Photo-Sensitive Moieties

D-DHP407 polymer functionalization with photo-sensitive moieties was performed through a water-based carbodiimide chemistry leading to the formation of amide bonds. To this purpose, TGA (>99%, Merck, Milan, Italy), CEA (Merck, Milan, Italy) and NBE (*endo* and *exo* mixture, Merck, Milan, Italy) were selected as grafting molecules to expose thiols, acrylates and norbornene moieties along the polymer backbone, respectively. Briefly, 1-ethyl-3-(3-dimethylaminopropyl) carbodiimide (EDC, TCI Europe, Zwijndrecht, Belgium) and N-hydroxysuccinimide (NHS, Merck, Milan, Italy) carbodiimide coupling reagents were first dissolved in ddH_2_O at 100 mg/mL and 50 mg/mL, respectively. Then, the activation of the –COOH groups belonging to TGA, CEA and NBE was conducted by dissolving the grafting molecules in the EDC/NHS solution at unitary –COOH:EDC/NHS molar ratio and adjusting the pH at 5. The reaction was carried on for 1 h at 4 °C, under vigorous stirring. Then, D-DHP407 solution (previously prepared at 18% *w/v* in ddH_2_O at 4 °C) was added to the reaction mixture to reach a final –NH/–COOH molar ratio of 1:20, while keeping the solution under stirring at 450 rpm. The grafting step was carried on for 6 h at RT after adjusting the pH at different values to investigate the influence of this parameter on the coupling efficiency. Specifically, pH values equal to 5, 7 and 9 were investigated for CEA and NBE grafting. Differently, TGA coupling was conducted for 1 h at 4 °C by setting an acidic pH (i.e., pH 4) according to the previously optimized procedure [34]. At the end of the grafting step, samples were put in dialysis (cut-off membrane 10–12 kDa, Merck, Milan, Italy) against ddH_2_O for 48 h at RT and in the dark. Finally, samples were freeze-dried using a Martin Christ ALPHA 2-4 LSC and stored under an inert atmosphere until use.

Hereafter, PEU exposing thiol, acrylate and norbornene groups will be referred to as S-DHP407_pH4, A-DHP407_pHX and NB-DHP407_pHX, respectively, where X stands for the pH used in the grafting step.

### 2.5. Chemical Characterization of Functionalized Poly(ether urethane)s

#### 2.5.1. Attenuated Total Reflectance Fourier Transform Infrared Spectroscopy

ATR-FTIR spectroscopy was conducted on all functionalized polymers to verify the integrity of the urethane bonds and to assess the success of the grafting procedure. D-DHP407 spectrum was also recorded and used as reference. Spectra were acquired by a Perkin Elmer Spectrum 100 (PerkinElmer, Inc., Waltham, USA)instrument equipped with an ATR accessory (UATR KRSS, PerkinElmer, Inc., Waltham, MA, USA) with diamond crystal and resulted from 32 scans in the range 4000–600 cm^−1^ with a resolution of 4 cm^−1^.

#### 2.5.2. Size Exclusion Chromatography

SEC analyses were performed to investigate whether the carbodiimide-mediated functionalization could affect polymer molecular weight. To this purpose, S-DHP407_pH4, A-DHP407_pHX and NB-DHP407_pHX samples were analyzed through an Agilent Technologies 1200 Series (Agilent Technologies Inc., Santa Clara, CA, USA) equipped with a Refractive Index (RI) detector and two Water Styragel columns (HR1 and HR4) conditioned at 55 °C and results compared to D-DHP407 ones. Polymers were first dissolved in the mobile phase (i.e., N,N-dimethylformamide (DMF, CHROMASOLV Plus, inhibitor free, for HPLC, 99.9%, Carlo Erba Reagents, Milan, Italy) added with 0.1% *w/v* LiBr (Merk, Milan, Italy)) at 2 mg/mL and then, filtered through a 0.45 μm syringe filter (poly(tetrafluoroethylene) membrane, Whatman). Number Average Molecular Weight (${\bar{M}}_{n}$), Weight Average Molecular Weight (${\bar{M}}_{w}$) and Polidispersity Index (D) were estimated by referring to a calibration curve based on poly(ethylene glycol) standards (range of peak molecular weight 4–200 kDa).

#### 2.5.3. Proton Nuclear Magnetic Resonance Spectroscopy

To further confirm the success of the functionalization procedure, Proton Nuclear Magnetic Resonance (^1^H NMR) spectroscopy was conducted on all functionalized samples and on D-DHP407 as control condition. In detail, 10 mg of PEU were first dissolved in 750 μL of deuterated dimethyl sulfoxide (d6-DMSO, Merck, Milan, Italy) and then, analyzed using a Bruker Avance NEO spectrometer (Bruker Biospin AG, Fällanden, Switzerland) equipped with a 11.74 T magnet (500 MHz ^1^H Larmor Frequency) and a Bruker multinuclear SMARTProbe probe. All the analyses were performed at 300 K using a Bruker SmartVT variable temperature control unit (BSVT) for temperature control. Each spectrum resulted from 32 scans, while the residual d6-DMSO proton signal at 2.5 ppm was used as reference for ^1^H chemical shift scale.

#### 2.5.4. Functional Group Quantification

Free thiol groups exposed along S-DHP407_pH4 polymeric chains were quantified through the colorimetric Ellman’s method by measuring the absorbance of yellow-colored products resulting from the reaction between PEU-SH and Ellman’s reagent (5,5′-dithio-bis-2-nitrobenzoic acid or DTNB, Merck, Milan, Italy) according to the previously reported protocol [34].

On the other hand, acrylate and norbornene moieties were quantified through ^1^H NMR spectroscopy by comparing the spectra of A-DHP407_pHX and NB-DHP407_pHX with those obtained analyzing the corresponding grafting molecules, i.e., CEA and NBE. Samples were prepared and analyzed as described in Section 2.5.3. To verify the repeatability of the functionalization process, the grafting condition giving the highest yield of functionalization was analyzed in triplicate. Results are reported as mean ± standard deviation.

### 2.6. Evaluation of Functionalized Poly(ether urethane) Thermo-Responsiveness at the Nano-Scale

Potential effects induced by polymer functionalization with photo-sensitive moieties on the temperature-driven chain capability to arrange into micelles were studied at the nano-scale through the measurement of the Critical Micellar Temperature (CMT) and the micelle hydrodynamic diameter. Analyses were performed on S-DHP407_pH4, A-DHP407_pHX and NB-DHP407_pHX polymers showing the highest number of functional groups (hereafter referred to with the acronyms S-DHP407, A-DHP407 and NB-DHP407, respectively) and on D-DHP407 as reference.

#### 2.6.1. Dynamic Light Scattering

Dynamic Light Scattering (DLS) measurements were performed using a Zetasizer Nano S90 (Malvern Instruments, Worcestershire, UK) instrument according to a recently published protocol [41,42,43]. Briefly, samples were prepared by dissolving the polymers in physiological saline solution (0.9% *w/v* NaCl) at 0.5% *w/v* concentration and equilibrated overnight at the test temperature. Analyses were performed at 25 °C, 37 °C and 45 °C and data were analyzed according to Pradal et al. [44]. The reported hydrodynamic diameters resulted from the average of three independently analyzed samples and they are expressed as mean ± standard deviation.

#### 2.6.2. Critical Micellar Temperature Evaluation

CMT values were estimated through UV/Vis spectroscopic analyses by using a micellization marker, i.e., 1,6-diphenyl-1,3,5-hexatriene (DPH, Merck, Milan, Italy) as previously reported [45]. Specifically, DPH was first dissolved in methanol at 0.4 mM and then, added at 10 μL/mL to samples prepared by dissolving the polymers at 0.1% *w/v* in physiological saline solution. Subsequently, samples were heated at a constant temperature increase (i.e., 1 °C/step) in the range 5–40 °C, equilibrated 5 min at each temperature and immediately analyzed through an UV/Vis spectrophotometer (Lambda 25, PerkinElmer, Inc., Waltham, MA, USA). Spectra were recorded in the 500–300 nm spectral range. Lastly, recorded absorbance intensity values at 356 nm were plotted against temperature and then, the CMT was defined as the temperature at which the absorbance sharply increased.

### 2.7. Thiol-Ene Hydrogel Preparation

Thiol-ene hydrogel formulations were prepared by dissolving the polymers at an overall 18% *w/v* polymeric concentration in Phosphate Buffered Saline (PBS, pH 7.4) solution. Furthermore, S-DHP407 and A-DHP407 or NB-DHP407 were mixed to achieve a 1:1 thiol-ene molar ratio. For both formulations, the photo-initiator Eosin Y disodium salt (EY, TCI chemicals, Zwijndrecht, Belgium) and the co-initiator (i.e., triethanolamine—TEOA—Merck, Milan, Italy and L-tyrosine—TYR—Merck, Milan, Italy for S-DHP407/A-DHP407 and S-DHP407/NB-DHP407, respectively) were dissolved in PBS at higher concentrations and then, different aliquots were added to the PEU mixtures to reach the final desired concentrations. Specifically, EY and TEOA were added at 1 mM and 7.5 mM, respectively, in S-DHP407/A-DHP407 solutions, while 0.5 mM and 0.1 mM EY and TYR concentrations, respectively, were used for S-DHP407/NB-DHP407 systems. Specifically, such photo-initiator and co-initiator concentrations resulted from a previous optimization aiming at maximizing the outcomes of the photo-irradiation step (data not reported). Co-initiator-free samples were also prepared according to the same protocol and compared to co-initiator-loaded formulations to investigate the role exerted by the co-initiator in the photo-crosslinking mechanism. Hydrogel solutions were kept at 4 °C until use to avoid micellization phenomena.

Hydrogel photo-irradiation was performed at 525 nm (i.e., green light) using a custom-made visible light-emitting source. Specifically, 200 μL of hydrogel solution were first pipetted in the solid state into a circular mold (10 mm diameter) positioned on a glass cover slip and then irradiated at 80,000 Lux for 10 min. Subsequently, exploiting the retained sample thermo-responsiveness, photo-irradiated hydrogel discs were collected and stored at 4 °C to allow their gel-to-sol transition before rheological characterization.

### 2.8. Rheological Characterization of Thiol-Ene Hydrogels

A stress-controlled rheometer (MCR302, Anton Paar GmbH, Graz, Austria) equipped with a 25 mm parallel plate geometry and a Peltier system for temperature control was used to rheologically characterize the developed thiol-ene formulations. Gel resistance to applied deformation was evaluated at 37 °C through strain sweep tests (10 Hz, strain range 0.01–500%), while gel viscoelastic properties were investigated by small amplitude oscillatory shear tests (frequency sweep tests performed within hydrogel linear viscoelastic range, frequency range 0.1–100 rad/s, strain 0.1%, temperature 25 °C, 30 °C and 37 °C). Photo-irradiated and control formulations, with or without co-initiator, were prepared as described in Section 2.7. A detailed description of system composition is reported in Table 1. Before each analysis, the sample was poured on the lower plate of the instrument in the sol state, heated at the selected temperature, kept in quiescent condition for 10 min to reach thermal stability and then tested.

### 2.9. Chemical Characterization of Photo-Crosslinked Thiol-Ene Hydrogels

The effective formation of covalent bonds between thiol and -ene (i.e., acrylate or norbornene) moieties after visible light exposure was assessed through the chemical characterization of photo-crosslinked thiol-ene hydrogels. Thiol-ene samples were also tested before photo-curing as control.

#### 2.9.1. Proton Nuclear Magnetic Resonance Spectroscopy

^1^H NMR spectroscopic analyses were conducted on the optimized thiol-ene formulations (as identified from rheological tests and prepared as reported in Section 2.7) according to the protocol described in Section 2.5.3. However, for these analyses, samples were prepared by dissolving the lyophilized powder at 40 mg/mL polymeric concentration in d6-DMSO. Not-irradiated thiol-ene samples were prepared following the same procedure and used as a control condition.

#### 2.9.2. Carbon Nuclear Magnetic Resonance Spectroscopy

Carbon Nuclear Magnetic Resonance (^13^C NMR) spectroscopy was conducted using a Bruker Avance NEO spectrometer equipped with a 11.74 T magnet (125.8 MHz ^13^C Larmor Frequency) and a Bruker multinuclear SMARTProbe probe (Bruker Biospin AG, Fällanden, Switzerland). Samples (photo-irradiated and not-photo-irradiated) were first prepared as described in Section 2.7, lyophilized and then, 60 mg of powder were dissolved in 750 μL of d6-DMSO. Spectra resulted from 1024 scans and the d6-DMSO signal at 39.5 ppm was used as reference for ^13^C NMR chemical shift scale.

### 2.10. Statistical Analysis

Statistical analysis was performed using GraphPad Prism 8.0 for MacOsX (GraphPad Software, La Jolla, CA, USA; www.graphpad.com, accessed on 15 December 2022). Two-way ANOVA analysis followed by Bonferroni’s multiple comparison test was used to compare results.

## 3. Results

### 3.1. Chemical Characterization of the Synthesized Poly(ether urethane)

The P407-based PEU (i.e., D-DHP407) used in this work resulted from an optimization of the synthetic procedure recently published by Laurano et al. [33]. Specifically, that work aimed at synthesizing a high molecular weight PEU bearing along its chains a huge amount of secondary amino groups as functional moieties suitable for further functionalization [33,34]. Therefore, for an in-depth discussion on the success of the synthesis and the exposure of secondary amines the reader could refer to this previously published paper [33]. Briefly, the comparison between P407 and DHP407 ATR-FTIR spectra clearly confirmed the successful synthesis of a PEU through the appearance of vibrational peaks ascribed to newly formed urethane bonds. Furthermore, ${\bar{M}}_{n}$ and ${\bar{M}}_{w}$ values measured for DHP407 turned out to be significantly higher compared to P407 (i.e., 20 kDa and 33 kDa vs. 9 kDa and 10 kDa) thus, proving the success of P407 chain extension leading to a higher molecular weight polymer. Moreover, the same chemical characterizations also proved the absence of degradation phenomena induced by the acidic treatment performed to remove Boc protecting groups, thus exposing secondary amines. Indeed, DHP407 and D-DHP407 ATR-FTIR spectra turned out to be completely overlapped, thus confirming the preservation of the urethane bond chemical integrity upon Boc removal. Lastly, secondary amino group quantification by the colorimetric Orange II Sodium Salt assay showed 4.5 × 10^20^ ± 1.8 × 10^19^ functional units/g of polymer.

### 3.2. D-DHP407 Functionalization with Photo-Sensitive Groups through Water-Based Carbodiimide Chemistry

**Figure 2:**
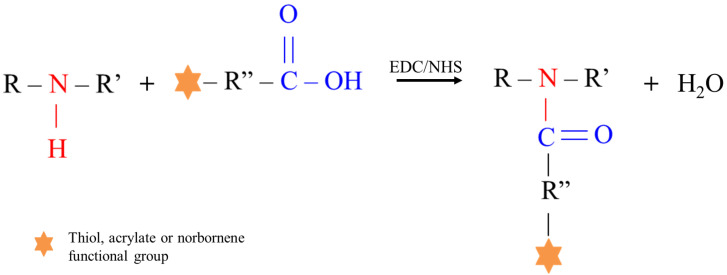
Schematic representation of carbodiimide-mediated tertiary amide bond formation between secondary amines and carboxylic acid groups exposed along the PEU and grafting molecule chains, respectively. The orange star stands for the exposed functional groups (i.e., thiols, acrylates or norbornene moieties).

In this work, the customized PEU bearing -NH groups was used as amphiphilic backbone to engineer multi-functional polymers (i.e., S-DHP407, A-DHP407 and NB-DHP407) for the preparation of injectable photo-click thiol-ene hydrogels responsive to temperature and visible light irradiation. Specifically, starting from a unique backbone, this study aimed at developing a versatile platform of dual-stimuli responsive PEUs by implementing environmental-friendly and low impactful functionalization procedures. More in detail, to impart PEU with responsiveness to visible light, the D-DHP407 polymeric backbone was functionalized with thiols, acrylates and norbornene groups, through the grafting of TGA, CEA and NBE molecules, respectively, by exploiting the versatility of a green water-based chemistry, i.e., the carbodiimide chemistry. However, carbodiimide chemistry is usually applied to react carboxylic acid groups with primary amino groups through the formation of secondary amides under mild conditions. Differently, in this work, this chemistry was explored as a coupling procedure to react –COOH group-containing molecules (i.e., TGA, CEA and NBE) with D-DHP407 bearing secondary amines, leading to the formation of tertiary amide bonds (Figure 2).

More in detail, carbodiimide chemistry consists of two steps: (i) –COOH group activation at acidic pH, and (ii) amide bond formation between –COOH and –NH functional moieties. The literature reports that the maximization of the grafting step can be achieved by adjusting the reaction pH at values higher than the pKa of the amino group-containing molecule (i.e., an alkaline pH is generally required) [46]. However, as –NH groups belonged to the custom-made D-DHP407 PEU characterized by a complex structure, it was difficult to experimentally determine their pKa. Therefore, the optimization of grafting conditions turned out to be an important step towards the maximization of the grafting yield.

#### 3.2.1. Influence of Grafting Reaction pH on the Degree of S-DHP407 Functionalization

The influence of reaction pH on the grafting of TGA molecules to D-DHP407 chains through carbodiimide chemistry has been recently reported by Laurano et al. [34]. In that work, only acidic pH values were investigated to optimize the grafting step as the preservation of thiol functionality during the functionalization procedure was of primarily importance, due to the highly labile stability of this photo-sensitive group. Indeed, by increasing the reaction pH from 4 to 7, the amount of exposed free -SH groups decreased. Hence, in this work TGA grafting to D-DHP407 polymeric chains was carried out by setting the reaction pH at 4. The comparison between S-DHP407 and D-DHP407 ATR-FTIR and ^1^H NMR spectra confirmed the success of the functionalization as previously reported [34]. Moreover, chromatographic analyses did not show any differences in the ${\bar{M}}_{n}$ and D values measured for both D-DHP407 and S-DHP407 (i.e., ${\bar{M}}_{n}$ 20 kDa, D 1.6). Lastly, the exposure of sulfhydryl groups was confirmed by Ellman’s colorimetric assay quantifying 1 × 10^19^ ± 1.3 × 10^18^ –SH groups/g of polymer.

#### 3.2.2. Influence of Grafting Reaction pH on the Degree of the -Ene Moiety Functionalization

To maximize the grafting of CEA and NBE to D-DHP407 chains and thus, the exposure of acrylate and norbornene double bonds, respectively, a similar approach to that used for thiomer design was adopted. However, differently from the pH values considered for TGA grafting optimization, in this study an alkaline pH was also included in the optimization of CEA and NBE grafting procedure, as no side-reactions affecting double bonds are known to occur at basic pH. Moreover, conversely to thiols, when treated in an acid environment, CEA- and NBE-double bonds could undergo heterolytic breaking, thus lowering the amount of available functional groups for further processing [47]. Lastly, in this work CEA and NBE binding was carried out at RT, while in our previous work TGA grafting was conducted at 4 °C to preserve thiol from disulfide bond formation, which is enhanced at higher temperatures. Differently, in this case the high stability of acrylate and norbornene groups ensures the retention of their functionality also when the grafting reaction is performed at 25 °C, with the additional advantage of leading to more thermodynamically favored interactions between –COOH and –NH functional groups.

**Figure 3:**
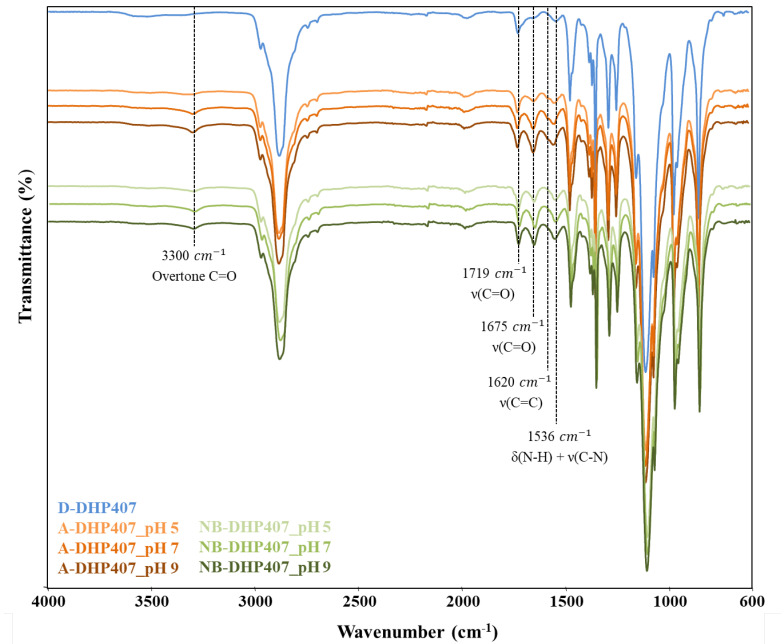
ATR-FTIR spectra of D-DHP407 (blue, control condition), A-DHP407_pHX (orange) and NB-DHP407_pHX (green). The increasing color intensity of the spectrum stands for the increasing reaction pH from 5 to 9. Dashed lines highlight the peaks attributed to the newly formed amide bonds and to C=C stretching vibration.

The success of the functionalization procedure was first assessed through ATR-FTIR spectroscopy, by comparing the spectra of A-DHP407_pHX and NB-DHP407_pHX samples with that of D-DHP407 (as control condition) as illustrated in Figure 3.

Irrespective of the reaction pH, a sharp increase in the intensity of the peaks ascribed to newly formed amide bonds was observed for A-DHP407_pHX and NB-DHP407_pHX samples if compared to the control (D-DHP407). In detail, significant differences were registered in the peaks at 1719 cm^−1^ and 1675 cm^−1^, which are attributed to carbonyl group (C=O) stretching vibrations [48]. Specifically, the band centred at 1675 cm^−1^ can be ascribed to the carbonyl groups of amide bonds thus confirming the formation of amide bonds between the –COOH groups of the grafting molecules and the secondary amines exposed along D-DHP407 polymeric chains. In addition, the appearance of a new band in the spectra of functionalized samples at 3300 cm^−1^ (ascribed to an overtone of C=O groups belonging to the amide bonds) further proved the successful functionalization. The absorption band at 1536 cm^−1^ can be attributed to the simultaneous stretching vibration of C–N bonds and bending vibration of N-H groups belonging to the urethane bonds. However, ATR-FTIR spectroscopy did not clearly highlight the appearance of peaks attributed to the C=C stretching vibration in the region 1550–1650 cm^−1^ due to the sharp intensity of the surrounding bands attributed to amide and urethane bonds. Indeed, only in A-DHP407_pHX spectra a shoulder at 1620 cm^−1^ was recognisable.

Concerning the pH-dependent efficiency of the grafting reaction, an increased intensity of the bands at 1675 cm^−1^ and 1536 cm^−1^ was observed for both A-DHP407_pHX and NB-DHP407_pHX PEUs by increasing the pH from 5 to 9. Hence, according to what theoretically expected, the coupling degree increased with increasing the pH value set during the grafting phase. These results were in contrast with our previous findings on the optimization of TGA grafting reaction, showing an opposite trend [34]. Indeed, although in the previous work this behaviour was attributed to the complex chemical structure of D-DHP407, which could modify the common reaction equilibrium of carbodiimides, in this case the pH-dependent CEA and NBE solubility in aqueous media must be considered as additional issue. In fact, by changing the pH from acid to alkaline values, the turbid CEA and NBE solutions at pH 5 became completely transparent at pH 9, suggesting a better solubilization of CEA and NBE molecules. As a consequence, at low pH values a low amount of soluble CEA and NBE molecules could be available to react with the -NH groups exposed along D-DHP407 chains, thus reducing the effective –NH/–COOH molar ratio. Hence, this different trend could be probably attributed to the more evident contribution of the pH-dependent CEA and NBE solubility rather than to the modified carbodiimide reaction equilibrium induced by the PEU structure.

On the other hand, irrespective of tested condition, chromatographic analyses did not evidence differences in the estimated molecular weights of A-DHP407_pHX and NB-DHP407_pHX compared to the D-DHP407 control (i.e., ${\bar{M}}_{n}$ 20 kDa, D 1.6).

**Figure 4:**
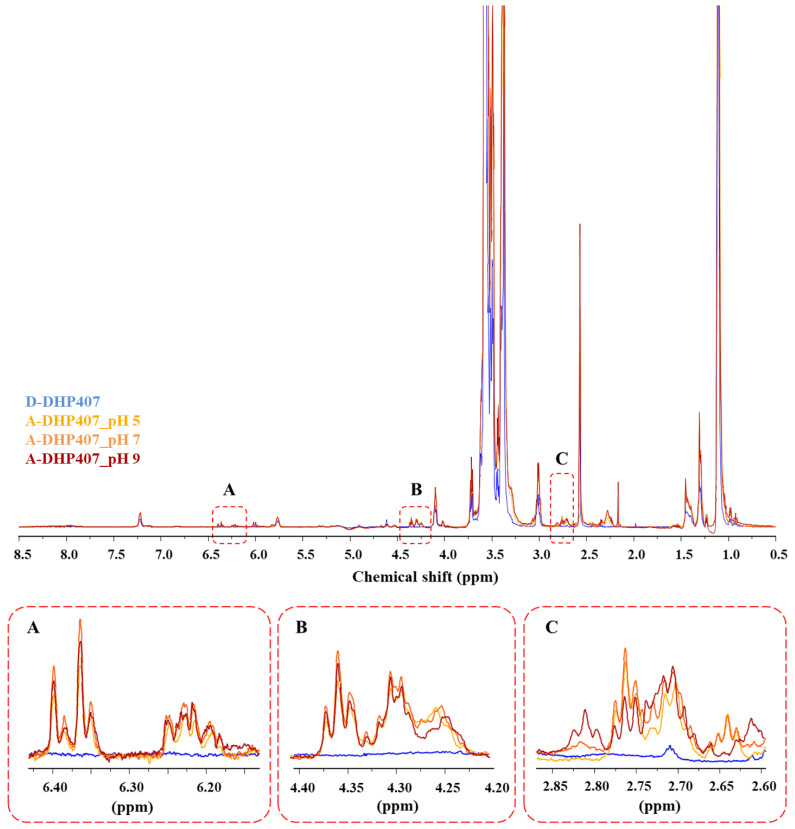
^1^H NMR spectra of D-DHP407 (blue, control condition) and A-DHP407_pHX (orange) samples. The increasing color intensity of the spectrum stands for the increasing reaction pH from 5 to 9. Magnified insert A highlights the signals attributed to the hydrogens involved in the double bonds of acrylate moieties in A-DHP407_pHX samples compared to D-DHP407 control PEU. Magnified inserts B and C highlight the signals ascribed to the resonances of the hydrogens involved in the –CH_2_ groups of CEA-grafted molecules. In magnified insert C, the triplet at 2.82 ppm present in A-DHP407_pH9 spectrum is produced by CEA complexation induced by alkaline pH values.

**Figure 5:**
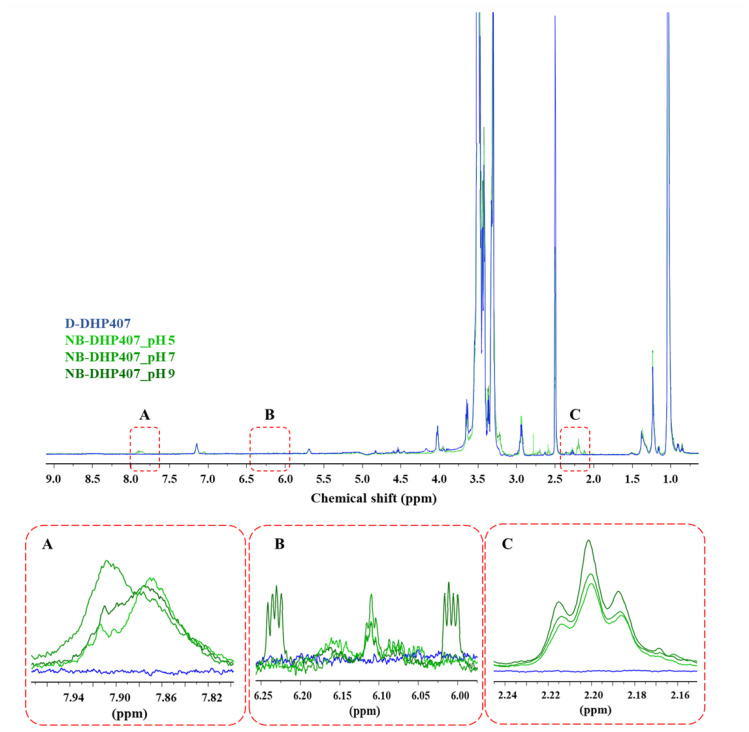
^1^H NMR spectra of D-DHP407 (blue, control condition) and NB-DHP407_pHX (green) samples. The increasing color intensity of the spectrum stands for the increasing reaction pH from 5 to 9. Magnified insert A highlights the signals attributed to the –NH groups of amides; magnified insert B highlights that both *endo* or *exo* forms of NBE were successfully grafted to PEU backbone with a pH-dependent yield (the peak at 6.1 ppm can be attributed to the *exo*-NBE, while the peaks at 6.0 and 6.25 ppm can be ascribed to the *endo*-NBE); magnified insert C evidences the triplet at 2.20 ppm due to the resonance of the aliphatic protons of NBE molecules.

To further confirm the success of CEA and NBE grafting to PEU secondary amino groups, A-DHP407_pHX and NB-DHP407_pHX samples were analyzed through ^1^H NMR spectroscopy and the acquired spectra were compared to D-DHP407 ^1^H NMR spectrum as control condition (Figure 4 and Figure 5).

Concerning A-DHP407_pHX spectra, irrespective of the pH set for the grafting reaction, new sets of signals appeared in the regions 2.6–2.7 ppm, 4.2–4.4 ppm and 6.2–6.4 ppm. Specifically, the first two sets (Figure 4, inserts B and C) were ascribed to the resonances of the hydrogens involved in the –CH_2_ groups of CEA-grafted molecules, while the bands within the 6.2–6.4 ppm range (Figure 4, insert A) were attributed to hydrogens involved in the double bonds [49]. Therefore, ^1^H NMR analyses confirmed the success of the acrylation procedure. As expected, differences were observed in the intensities of these peaks based on the reaction pH. In particular, the intensities of these bands increased from A-DHP407_pH5 to A-DHP407_pH9, in agreement with ATR-FTIR results. However, A-DHP407_pH9 spectrum showed the appearance of a new triplet at 2.82 ppm, probably ascribed to CEA complexation induced by alkaline pH values (Figure 4, insert C).

^1^H NMR spectra of NB-DHP407_pHX (Figure 5) showed similar results, further increasing the consistency of the previously formulated hypotheses. Indeed, irrespective of the pH used for the grafting reaction, also NB-DHP407_pHX spectra showed the appearance of new sets of signals that confirmed the presence of NBE and the success of norbornene grafting to D-DHP407 chains. Specifically, the band at 7.85–7.90 ppm (Figure 5, insert A) could be attributed to the –NH groups of amides, as reported by Qin et al. [50]. Moreover, a new band also appeared at 2.2 ppm (Figure 5, insert C) with an intensity slightly more higher in NB-DHP407_pH9 compared to NB-DHP407_pH5 and NB-DHP407_pH7. Specifically, this triplet can be attributed to the resonance of the aliphatic protons of NBE molecules in agreement with McOscar et al. [51]. Moreover, the analysis of the spectra within 5.9 and 6.3 ppm (Figure 5, insert B) evidenced that both *endo* and *exo* forms of NBE were successfully grafted to PEU backbone with a pH-dependent yield [52]. Indeed, the peak at 6.1 ppm attributed to the *exo*-NBE double bond protons was present in all NB-DHP407_pHX spectra, with higher intensity in NB-DHP407_pH7. Conversely, the peaks at 6.0 and 6.25 ppm ascribed to the *endo*-NBE double bond protons were present only in NB-DHP407_pH9 spectrum. Hence, although both ATR-FTIR and ^1^H NMR analyses (Figure 3 and Figure 5, respectively) proved the success of the grafting in all tested pH conditions, the set of signals ascribed to the protons involved in the C=C double bonds was remarkably evident only in NB-DHP407_pH9 spectrum.

Lastly, ^1^H NMR spectroscopy was also exploited to quantify acrylate and norbornene functional groups by measuring the area subtended by the signals at 6.4 ppm and between 6.0 and 6.25 ppm, respectively, and comparing them with the corresponding area calculated in CEA and NBE spectra.

The amount of grafted CEA was 1.8 × 10^19^, 2.6 × 10^19^ and 2.7 × 10^19^ units/g of A-DHP407_pH5, A-DHP407_pH7 and A-DHP407_pH9, respectively. Hence, although very similar results were obtained, pH 9 was the optimal reaction pH to maximize CEA grafting. However, due to the appearance of the previously discussed additional peak at 2.82 ppm in the ^1^H NMR spectrum of A-DHP407_pH9, it was not considered for further optimization. Conversely, A-DHP407_pH7 was considered as optimal and further investigated to verify the repeatability of the process. The quantification of acrylate functional groups grafted on samples belonging to three different A-DHP407_pH7 batches gave a mean value of 2.6 × 10^19^ ± 1.5 × 10^18^ acrylate groups/g of polymer.

On the other hand, the grafted NBE amounts turned out to be 6.2 × 10^16^, 7.4 × 10^16^ and 8.3 × 10^17^ units/g of NB-DHP407_pH5, NB-DHP407_pH7 and NB-DHP407_pH9, respectively. Therefore, functional group quantification definitely confirmed that similar results were obtained by carrying out the grafting reaction at pH 5 and 7, while pH 9 was able to graft an almost one order of magnitude higher amount of NBE to D-DHP407 polymer chains. Hence, this condition was further investigated to verify the repeatability of the process with a mean value of norbornene groups/g of polymer equal to 8.1 × 10^17^ ± 2.6 × 10^16^ as quantified by considering three independent carbodiimide procedures.

Hereafter, the acronyms A-DHP407 and NB-DHP407 will be used to refer to PEUs functionalized according to the optimized protocols, i.e., by using a grafting reaction pH equal to 7 and 9, respectively.

### 3.3. Evaluation of the Thermo-Responsiveness of Functionalized Poly(ether urethane)s

P407 was selected as PEU building block due to its amphiphilic nature, which ensures the thermo-responsiveness of the resultant water-based formulations as assessed for similar P407-based polymers [42,45]. Furthermore, such feature was not altered by the acidic treatment performed on DHP407 to expose secondary amino groups [33]. In this work, the influence of D-DHP407 functionalization with TGA, CEA and NBE molecules on the temperature-dependent micellar structure organization was investigated through DLS measurements and by estimating the CMT values of S-DHP407, A-DHP407 and NB-DHP407 aqueous solutions compared to D-DHP407 samples with similar composition.

Figure S1 reports the intensity patterns acquired through DLS measurements at 25 °C for D-DHP407, S-DHP407, A-DHP407 and NB-DHP407 aqueous solutions at 0.5% *w/v* concentration. Clearly organized structures were not detectable for D-DHP407, S-DHP407 and A-DHP407 samples at this temperature due to the high system instability ascribed to continuous micelle/cluster aggregation and disaggregation. Differently, three well-defined peaks were detected in NB-DHP407 intensity patterns at 15.2 ± 1.4 nm, 59.2 ± 1.0 nm and 528.0 ± 57.2 nm, which can be attributed to unimers, micelles and aggregates, respectively [31]. Hence, NB-DHP407 systems showed higher stability compared to D-DHP407, S-DHP407 and A-DHP407-based ones, probably ascribed to the presence of a markedly hydrophobic component (i.e., NBE molecules) in the PEU backbone, which could favor chain arrangement into micelles still at RT. Upon heating up to physiological temperature and 45 °C (data not reported), a well-defined double peak appeared in all the registered intensity patterns, with average hydrodynamic diameters ascribable to single micelles and aggregates, proving that micelle nucleation and clustering proceeded upon temperature increase [34]. Hence, as expected, D-DHP407 functionalization with TGA, CEA and NBE molecules did not affect polymer capability to arrange into micelles, being this responsiveness ensured by the building blocks not involved in such polymer modification.

**Figure 6:**
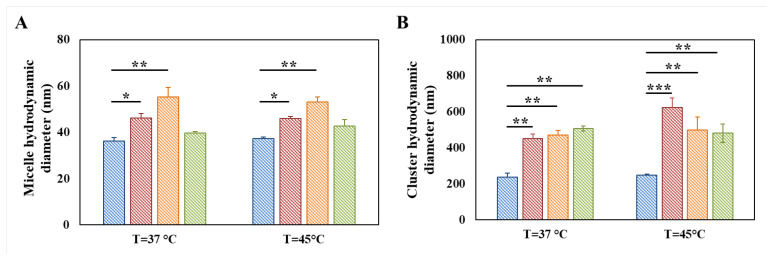
Average micelle (**A**) and cluster (**B**) hydrodynamic diameters measured for D-DHP407 (blue), S-DHP407 (red), A-DHP407 (orange) and NB-DHP407 (green) samples at 0.5% *w/v* concentration and different temperatures (i.e., 37 °C and 45 °C). Statistical significance: 0.0001 < *p* < 0.001 = ***, 0.001 < *p* < 0.01 = **, 0.01 < *p* < 0.5 = *.

However, irrespective of grafted molecules, both micelles and clusters showed statistically significant higher hydrodynamic diameters in functionalized PEU samples compared to D-DHP407-based ones, at both 37 and 45 °C (Figure 6). Specifically, in the case of TGA grafting, such dimensional increase was attributed to the formation of a thicker hydrated shell around micelles and clusters induced by the presence of thiol groups [34]. Concerning A-DHP407 formulations, the measured micelle average hydrodynamic diameter was almost two-fold higher than that reported for single D-DHP407 micelles. These observations could find a double potential explanation: (i) in A-DHP407 samples, micelles were not present as single structures at 37 °C but in the form of small aggregates; (ii) being acrylate functional moieties characterized by a hydrophobic nature they could be internalized in the hydrophobic micelle core thus resulting in the formation of bigger micelles [41]. However, in this second case, acrylate groups would have been only partially available during the photo-induced reaction with thiol groups. The groundlessness of this second hypothesis will be thoroughly demonstrated in the following paragraph, proving that the entire amount of double bonds present in the systems will be involved in the photo-crosslinking reaction. Therefore, these observations could be reasonably attributed to the formation of small micelle aggregates at 37 °C rather than to the formation of single micelles with larger average hydrodynamic diameters. This hypothesis was also further supported by considering that the grafting of CEA to PEU backbone increased the hydrophobic content of the system and thus, polymeric chains more easily aggregated reducing the interfacial area in contact with water molecules. Lastly, further heating up to 45 °C did not produce a further increase in the average hydrodynamic diameters of micelles and clusters with values measured to be 53.2 ± 2.1 nm and 497.6 ± 72.1 nm, respectively.

Considering NB-DHP407 formulations, temperature increase up to 37 °C resulted in the disappearance of the peak attributed to unimers thus, suggesting the complete chain arrangement in micellar structures. However, differently from S-DHP407 and A-DHP407, the average hydrodynamic diameter of NB-DHP407 micelles measured at 37 °C and 45 °C (i.e., 39.7 ± 0.6 nm and 42.8 ± 2.8 nm, respectively) was lower than at 25 °C (i.e., 59.2 ± 0.1 nm). These observations can be attributed to the progressively more evident phase separation occurring between micelles and water molecules (dehydration of PEO blocks) upon temperature increase, with water molecules moving towards the interstitial space among micelles [53]. Regarding cluster diameter, similar values were measured for NB-DHP407 samples at 37 °C and 45 °C, which turned out to be statistically different compared to control samples.

Lastly, the temperature-driven micelle organization was also investigated through UV/Vis spectroscopic analyses exploiting a micellization marker able to give an intense peak at 356 nm only when dispersed in a hydrophobic environment, such as the micelle core. The temperature at which the peak at 356 nm appeared was registered at 24 °C and 28 °C for not-functionalized and functionalized PEUs, respectively. Hence, the grafting of TGA, CEA and NBE molecules to D-DHP407 initially slightly hampered chain arrangement into micellar structures. In the case of S-DHP407, this observation was attributed to an increased hydrophilic content of the system, leading to stronger and prolonged interactions of polymeric chains with the surrounding water molecules. Conversely, the delayed chain organization in A-DHP407 and NB-DHP407 samples can be explained considering the presence of pendant hydrophobic lateral chains showing remarkable steric hindrance. Despite such initial differences, all considered samples were able to form micelles, in accordance with DLS results, and the CMT values were estimated to be 23.3 °C, 25.6 °C, 24.0 °C and 24.2 °C for D-DHP407, S-DHP407, A-DHP407 and NB-DHP407 samples, respectively.

### 3.4. Design of Thermosensitive and Photo-Click Thiol-Ene Formulations

Thiol-ene S-DHP407/A-DHP407 and S-DHP407/NB-DHP407 gelling systems were obtained by mixing the polymers at 1:1 functional group molar ratio (18% *w/v* polymer concentration). The thermo-responsiveness was exploited to achieve a gel state at physiological temperature, while the sensitivity to visible light was investigated to reinforce the gel network through the formation of thiol-ene bonds without the need of additional crosslinking agents. However, despite the achievement of stronger gels upon photo-irradiation, both formulations were able to preserve their thermo-responsiveness, thus allowing a reverse gel-to-sol transition at 4 °C. Specifically, such powerful feature for biomedical applications was also exploited to thoroughly study the process of gel formation and to investigate potential improvements in the rheological properties upon photo-irradiation. Moreover, the role of the co-initiator as well as the influence of the –ene moieties on the photo-crosslinking mechanism were investigated exploiting the same approach. Lastly, the success of the photo-crosslinking mechanism was definitely proved by chemically characterizing S-DHP407/A-DHP407 and S-DHP407/NB-DHP407 samples upon exposure to green light.

#### 3.4.1. Thiol-Acrylate Photo-Click Hydrogels

**Figure 7:**
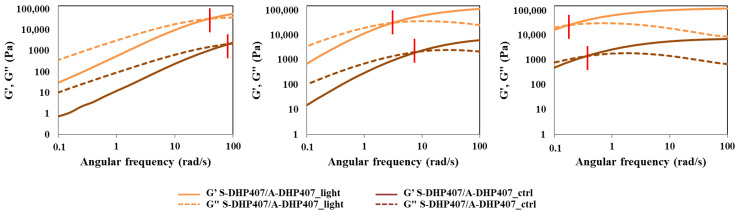
Frequency sweep tests conducted at 25 °C, 30 °C and 37 °C (from left to right) on S-DHP407/A-DHP407 formulations before (dark orange) and after (light orange) exposure to green light (525 nm, 80,000 Lux for 10 min). Continuous and dashed lines represent the storage (G’) and loss (G”) moduli, respectively, while the red bars identify the G’/G’’ crossover frequency. For higher clarity, G’ and G” values of control samples were divided by a factor of 10.

The temperature-driven PEU chain arrangement into an organized network was first studied through frequency sweep tests performed at 25 °C, 30 °C and 37 °C on not-irradiated S-DHP407/A-DHP407 formulation (i.e., S-DHP407/A-DHP407_ctrl) and then, compared to the same system subjected to light exposure (i.e., S-DHP407/A-DHP407_light) (Figure 7). According to the relationships between the storage (G’) and the loss (G”) moduli defining the physical state of the sample [54], both formulations were in the sol state at 25 °C, in a biphasic state at 30 °C and in a gel (although not fully developed) phase at 37 °C. Thus, these tests demonstrated the temperature-driven gelling mechanism and proved the retained capability of photo-irradiated polymeric chains to arrange into organized structures when subjected to a temperature increase.

However, at each analyzed temperature, the G’/G” crossover frequency (ω_crossover_) of S-DHP407/A-DHP407_light was at remarkable lower frequency values compared to not-irradiated samples. At 25 °C (sol state) and 30 °C (bi-phasic state), the lower crossover frequency of S-DHP407/A-DHP407_light systems compared to control samples (i.e., 35 rad/s vs. 90 rad/s and 4 rad/s vs. 8 rad/s, respectively) suggested effective thiol-acrylate linking reactions upon irradiation. At 37 °C, such double gel formation mechanism (i.e., temperature and visible light induced mechanism) led to the achievement of a more developed gel state for S-DHP407/A-DHP407_light compared to the control (i.e., ω_crossover_ equal to 0.18 rad/s vs. 0.35 rad/s), further suggesting the successful Vis-light initiated chain reaction, as a consequence of thiol-acrylate interactions. Although Vis-light irradiated hydrogels retained their capability to undergo gel-to-sol transition at low temperatures, the light-induced covalent bonds formed between functional groups permanently changed the chain structure. Such chemical bonds further stabilized the hydrogels formed by heating to 37 °C, which assumed a more organized structure (i.e., lower viscous component contribution) compared to not-photo-irradiated systems.

The comparison of strain sweep tests performed at 37 °C on S-DHP407/A-DHP407 samples before and after Vis-light exposure further supported previous findings (Figure S2). In fact, the difference between the storage and loss moduli calculated at 0.01% strain (i.e., Δ(G’ − G”)_@0.01% strain_) for S-DHP407/A-DHP407_light samples was substantially higher compared to control samples, i.e., 8082 Pa vs. 3880 Pa, meaning that photo-irradiated hydrogels were stronger gels than control samples at 37 °C. Moreover, S-DHP407/A-DHP407_light formulations showed a higher critical deformation (i.e., limit of the linear viscoelastic region) compared to not-irradiated gels (i.e., 18.6% vs. 11.6%), probably because the formation of covalent bonds among polymer chains provided the resulting gel network with an improved capability to withstand applied strains.

#### 3.4.2. Thiol-Norbornene Photo-Click Hydrogels

**Figure 8:**
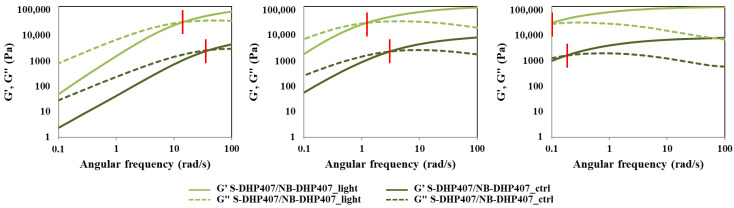
Frequency sweep tests conducted at 25 °C, 30 °C and 37 °C (from left to right) on S-DHP407/NB-DHP407 formulations before (dark green) and after (light green) exposure to green light (525 nm, 80,000 Lux for 10 min). Continuous and dashed lines represent the storage (G’) and loss (G”) moduli, respectively, while the red bars identify the G’/G’’ crossover frequency. For higher clarity, G’ and G” values of control samples were divided by a factor of 10.

Similarly to S-DHP407/A-DHP407 hydrogels, also S-DHP407/NB-DHP407 formulations were rheologically characterized before and after light exposure at 525 nm to investigate changes in hydrogel network organization and mechanical behavior due to visible-light initiated photo-click thiol-ene reactions. Figure 8 reports the trends of G’ and G’’ as measured during frequency sweep tests performed at 25 °C, 30 °C and 37 °C.

According to previous findings, also S-DHP407/NB-DHP407 formulations showed a temperature-induced sol-to-gel transition and the retained capability of photo-irradiated samples to re-arrange into organized structures (hydrogels) when subjected again to temperature increase. At each analyzed temperature, ω_crossover_ of S-DHP407/NB-DHP407_light was at remarkable lower frequency values compared to S-DHP407/NB-DHP407_ctrl, i.e., 15 rad/s vs. 41 rad/s, 1.4 rad/s vs. 3.0 rad/s and <0.1 rad/s vs. 0.18 rad/s at 25 °C, 30 °C and 37 °C, respectively. These results suggested that at each tested temperature, the elastic behavior of S-DHP407/NB-DHP407_light systems prevailed over the viscous behavior for a wider frequency range compared to the control, thus proving the successful Vis-light initiated chain coupling as a consequence of thiol-norbornene interactions. Indeed, although photo-irradiated hydrogels preserved their gel-to-sol reversibility at low temperatures, light-induced covalent bonds formed between functional groups permanently changed the chain structure. Thus, when the systems were subjected to controlled heating, crosslinked chains were probably able to more quickly arrange into an organized network.

Furthermore, results from strain sweep tests performed at 37 °C on S-DHP407/NB-DHP407 samples before and after Vis-light exposure indicated that they became mechanically stronger upon photo-irradiation (Figure S3). Indeed, Δ(G’ − G”)_@0.01% strain_ calculated for S-DHP407/NB-DHP407_light samples turned out to be almost two-fold higher compared to the control (i.e., 10,206 Pa vs. 5345 Pa), meaning that photo-irradiated hydrogels were significantly more developed at 37 °C. Moreover, an increase in critical deformation was also observed in S-DHP407/NB-DHP407_light compared to S-DHP407/NB-DHP407_ctrl (from 7.2% to 11.6%), which can be correlated to an enhanced capability of photo-irradiated systems to withstand applied deformations, because of the formation of bridges among the polymeric chains.

#### 3.4.3. The Role Exerted by the Co-Initiator in the Thiol-Ene Photo-Crosslinking Process

Beneficial effects on the initiation efficiency potentially deriving from the addition of a co-initiator were investigated for both S-DHP407/A-DHP407 and S-DHP407/NB-DHP407 formulations. Indeed, the literature reports the need to combine dye photo-initiators, such as Eosin Y, with co-initiators capable of electron transfer with excited states to generate free radicals [40,55]. Radicals formed by co-initiators are generally more reactive to initiate the polymerization of monomers [55]. Among the investigated co-initiators, amines are the most exploited ones [56]. Based on previous studies, TEOA [39] and TYR [40] were selected as co-initiators for thiol-acrylate and thiol-norbornene systems, respectively.

Irrespective of the considered thiol-ene formulation, the addition of a co-initiator did not alter their retained thermo-responsiveness upon photo-irradiation. Indeed, both not-irradiated and irradiated systems underwent a temperature-driven sol-to-gel transition as suggested by ω_crossover_ shifts towards lower frequencies upon temperature increase (Figure S4A,B). Furthermore, the comparison between ω_crossover_ measured for photo-irradiated and not-photo-irradiated systems proved the successful thiol-acrylate and thiol-norbornene photo-activated reactions in the presence of TEOA and TYR, respectively (Table 2).

Indeed, at each tested temperature, photo-irradiated samples showed a prevalent elastic behavior with respect to control samples. These evidences were further supported by strain sweep tests performed at physiological temperature (Figure S4C), showing differences between G’ and G” at 0.01% strain remarkably higher for photo-irradiated hydrogels compared to their corresponding control. Moreover, higher strain resistance to applied deformation was measured for S-DHP407/A-DHP407_TEOA_light compared to the control, meaning that photo-irradiation resulted in a more crosslinked network. Conversely, no significant differences were observed for thiol-norbornene formulations after photo-irradiation.

**Figure 9:**
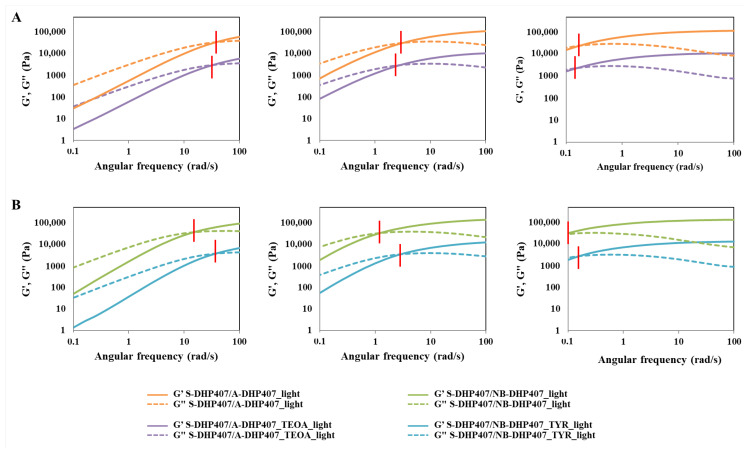
Frequency sweep tests performed at 25 °C, 30 °C and 37 °C (from left to right) on S-DHP407/A-DHP407 hydrogels (**A**) with and without TEOA (violet and orange, respectively) and S-DHP407/NB-DHP407 formulations (**B**) with and without TYR (light blue and green, respectively) after exposure to visible light (525 nm, 80,000 Lux, 10 min). For higher clarity, G’ and G” values of co-initiator-loaded formulations were divided by a factor of 10.

To investigate whether the addition of a co-initiator was effectively able to further improve the photo-activated initiation reaction, G’ and G” profiles acquired for co-initiator-free and co-initiator-loaded formulations after light irradiation were compared (Figure 9).

At each tested temperature, TEOA addition in thiol-acrylate formulations resulted in ω_crossover_ shifts towards lower frequencies compared to S-DHP407/A-DHP407 ones (Figure 9A), thus confirming an enhanced TEOA-mediated thiol-acrylate photo-click reaction. However, upon photo-irradiation, TEOA addition decreased the critical deformation of S-DHP407/A-DHP407_TEOA gels (i.e., 11.6%) compared to S-DHP407/A-DHP407 (i.e., 18.6%) as measured through strain sweep tests at 37 °C. This result could be ascribed to a mixed-mode photo-polymerization mechanism (i.e., the combination of step-growth and chain-growth polymerization processes), which is enhanced by the addition of a co-initiator. Indeed, when the -ene moiety in thiol-ene systems is represented by acrylates, their susceptibility to homopolymerization results in the formation of more heterogeneous networks characterized by lower capability to withstand applied deformations compared to bio-orthogonal ones [57,58,59].

An opposite situation was observed for thiol-norbornene formulations (Figure 9B). At each analyzed temperature, remarkably different ω_crossover_ frequencies were measured between photo-irradiated thiol-norbornene formulations not-containing or containing TYR. Specifically, TYR-free S-DHP407/NB-DHP407 samples showed prevalent elastic behaviour at lower frequency compared to TYR-containing formulations. These observations suggested that the addition of a co-initiator probably decreased the formation of covalent thiol-norbornene bonds, which in turn resulted in the formation of less developed gels at 37 °C and less elastic sol systems at 25 °C and 30 °C. Indeed, in the case of thiol-norbornene photo-irradiation, the reaction should proceed at unitary functional group molar ratio. Hence, the addition of a co-initiator, which could mainly increase the amount of double bond-centred radicals, did not improve thiol-norbornene reaction efficiency. Conversely, it could cause an impaired thiol and norbornene radical formation, potentially leading to norbornene double bond consumption with other reactive species present in the mixture instead of thiols. Thus, these results further supported the theory on step-growth polymerization as the only reaction mechanism occurring between thiol and norbornene functional groups when exposed to Vis-light [58]. Moreover, these hypotheses were confirmed by the worsened mechanical properties registered for S-DHP407/NB-DHP407_TYR systems compared to TYR-free ones, i.e., lower Δ(G’ − G”)_@0.01% strain_ (8950 Pa vs. 10,205 Pa) and critical deformation (7.2% vs. 11.6%).

As a result, the optimal formulations to improve gel mechanical and residence features compared to physical systems were found to be TEOA-loaded S-DHP407/A-DHP407 and TYR-free S-DHP407/NB-DHP407 systems.

#### 3.4.4. Chemical Characterization of Thiol-Ene Photo-Crosslinked Hydrogels

The success of green light-induced thiol-ene reactions was definitely assessed by chemically characterizing both S-DHP407/A-DHP407_TEOA_light and S-DHP407/NB-DHP407_light formulations. Not-irradiated systems were analyzed for comparison. ATR-FTIR spectroscopy and SEC analyses (data not reported) demonstrated the absence of degradation phenomena induced by light exposure but, no clear evidences ascribable to the successful photo-crosslinking were detected. Indeed, ATR-FTIR spectra before and after photo-irradiation were completely overlapped with no additional bands ascribed to newly formed bonds (i.e., C-S linkages in the 800–600 cm^−1^ spectral range [60]). On the other hand, slight differences in the peak molecular weight values were registered compared to control formulations, but ${\bar{M}}_{n}$ and ${\bar{M}}_{w}$ were almost comparable to not-irradiated systems. Hence, to thoroughly investigate the simultaneous thiol and double bond functional group consumption upon Vis-light exposure, ^1^H NMR and ^13^C NMR analyses were performed on S-DHP407/A-DHP407_TEOA and S-DHP407/NB-DHP407 formulations.

**Figure 10:**
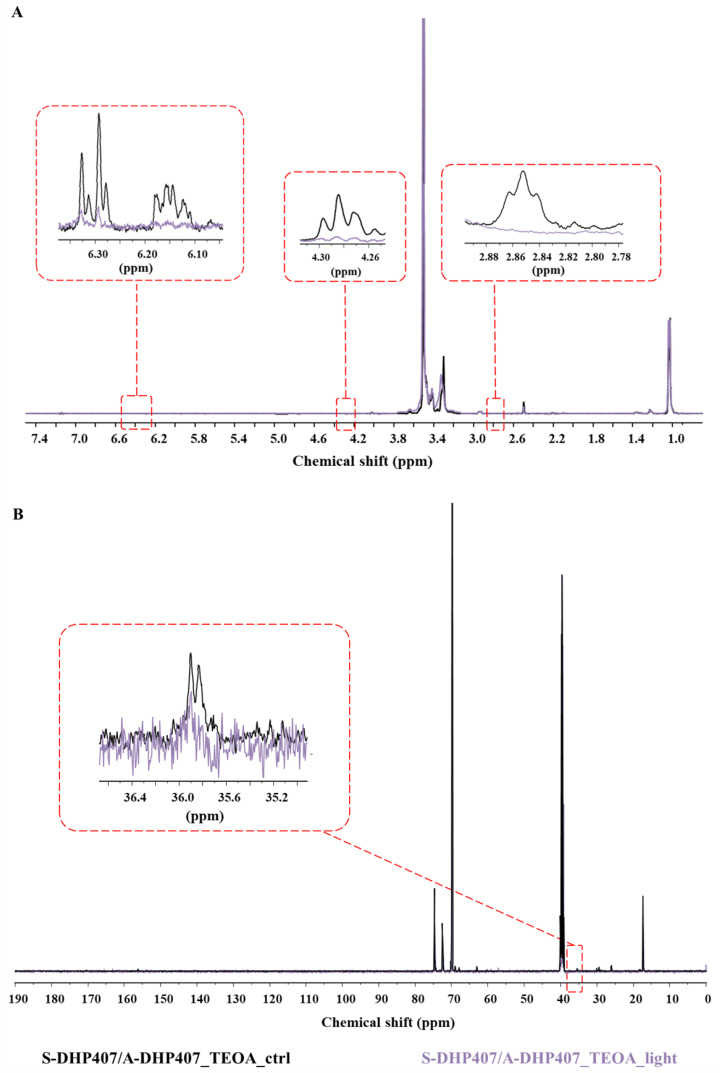
^1^H NMR spectra of S-DHP407/A-DHP407_TEOA_light (violet) and S-DHP407/A-DHP407_TEOA_ctrl (black) samples (**A**). Magnified inserts highlight differences in the bands at 2.8 ppm and 4.3 ppm corresponding to the resonances of protons in -CH_2_ groups of acrylate-bearing chains, and the set of signals at 6.1–6.3 ppm ascribed to protons involved in the double bonds. ^13^C NMR spectra of S-DHP407/A-DHP407_TEOA_light (violet) and S-DHP407/A-DHP407_TEOA_ctrl (black) samples (**B**). Magnified insert evidences changes in the signals at 35.8 ppm and 35.9 ppm, due to the methylene carbons of TGA molecules showing free –SH moiety or converted into S-S bonds, respectively.

Concerning thiol-acrylate systems, the comparison of ^1^H NMR spectra before and after light exposure showed complete disappearance of the bands at 2.8 ppm and 4.3 ppm corresponding to the resonances of protons in -CH_2_ groups of acrylate-bearing chains (Figure 10A). These observations could be attributed to a changed acrylate chemical environment upon light exposure, which would suggest their responsiveness to visible-light irradiation. Moreover, such hypothesis was further supported by the remarkable intensity decrease of the set of signals at 6.1–6.3 ppm ascribed to protons involved in the double bonds [49]. However, this intensity reduction was not complete, meaning that residual unreacted acrylate groups were still present in photo-irradiated hydrogels. On the other hand, evidences of thiol consumption as a consequence of thiol-acrylate hydrogel photo-irradiation were provided by ^13^C NMR spectroscopy (Figure 10B). Indeed, S-DHP407/A-DHP407_TEOA_ctrl spectrum showed two distinct peaks at 35.8 ppm and 35.9 ppm, corresponding to the methylene carbons of TGA molecules showing free –SH moiety or converted into S-S bonds, respectively [34]. Conversely, S-DHP407/A-DHP407_TEOA_light spectrum showed only a low intensity peak at 35.9, i.e., the band ascribed to the presence of S-S bonds. Hence, we could assume that all available free thiols were consumed upon irradiation, as a consequence of their reaction with acrylate groups. Moreover, the reduction of the band at 35.9 ppm suggested that also disulfide bonds were involved in the photo-irradiation process, as they typically undergo radical-mediated exchange reactions [61]. Such potentially available thiols were not considered in the quantification of free thiols, leading to an irrelevant imbalance in the thiol and acrylate molar ratio.

Thus, NMR spectroscopy confirmed the successful reaction between thiol and acrylate groups, as the intensity of the characteristic bands ascribed to acrylate double bonds and thiol groups both decreased in S-DHP407/A-DHP407_TEOA_light spectra. However, NMR analysis could not provide any information on acrylate homopolymerization. Conversely, it was possible to exclude mutual interactions between thiols upon photo-irradiation, because the band attributed to disulfide bonds showed a decreased intensity.

**Figure 11:**
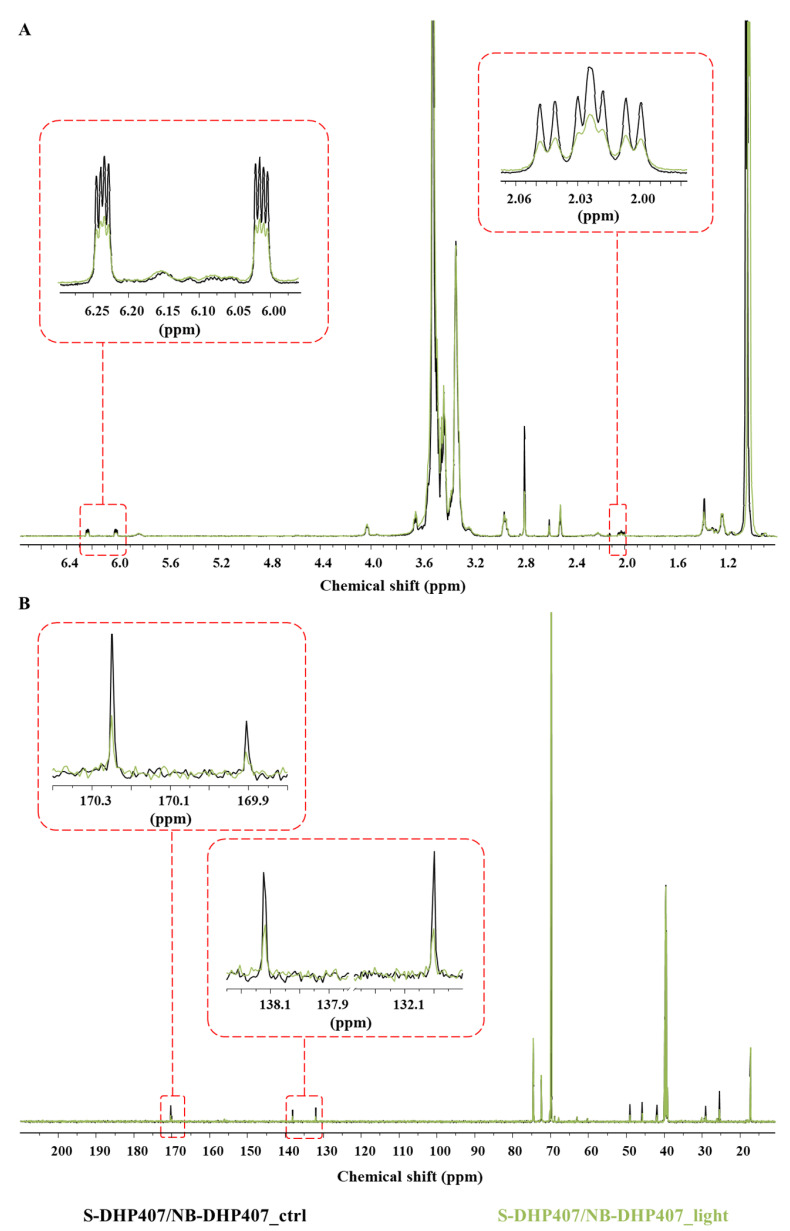
^1^H NMR spectra of S-DHP407/NB-DHP407_light (light green) and S-DHP407/NB-DHP407_ctrl (black) samples (**A**). Magnified inserts highlight differences in the set of signals between 2.0 ppm and 2.05 ppm corresponding to the aliphatic protons of norbornene molecules, and the bands at 6.0–6.2 ppm due to protons involved in norbornene double bonds. ^13^C NMR spectra of S-DHP407/NB-DHP407_light (light green) and S-DHP407/NB-DHP407_ctrl (black) samples (**B**). Magnified inserts evidence changes in the signals at 132 ppm and 138.2 ppm ascribed to the carbons of norbornene double bonds, the peak at 170.3 ppm ascribed to the resonance of the carbonyl group involved in the amide bond between PEU and TGA preserving the free –SH groups, and the band at 169.9 ppm attributed to the carbonyl group involved in the amide bonds formed with TGA molecules converted in the S-S form.

Regarding thiol-norbornene formulations (Figure 11A), the comparison of S-DHP407/NB-DHP407_light and control ^1^H NMR spectra showed a remarkable reduction of the set of signals between 2.0 ppm and 2.05 ppm, corresponding to the aliphatic protons of norbornene molecules. These observations could be attributed to a changed norbornene chemical environment upon light exposure, suggesting their visible-light induced responsiveness. These observations were further supported by the substantial intensity decrease of the bands at 6.0–6.2 ppm in S-DHP407/NB-DHP407_light spectrum ascribed to protons involved in norbornene double bonds [62]. However, no complete disappearance of these signals was observed, meaning that residual unreacted norbornene groups were still present in photo-irradiated hydrogels. This incomplete double bond consumption could be attributed to (i) the steric hindrance and high rigidity of norbornene molecules which could limit their exposure towards the interstitial space, thus making them less available for interaction with thiols, and (ii) the impaired functional group consumption in the case of disulfide bond formation as by-products.

This last-mentioned hypothesis was further investigated through ^13^C NMR spectroscopy (Figure 11B), showing the presence of residual norbornene double bonds as evidenced by the reduced band intensities at 132 ppm and 138.2 ppm. Moreover, the presence in the S-DHP407/NB-DHP407_light spectrum of a residual peak at 170.3 ppm, ascribed to the resonance of the carbonyl group involved in the amide bond between PEU and TGA preserving the free –SH groups [34], suggested that there were still thiol groups available for reaction. In addition, the slight reduction in the band at 169.9 ppm, attributed to the carbonyl group involved in the amide bonds formed with TGA molecules converted in the S-S form [34], highlighted the partial consumption of disulfide bonds through radical-mediated exchange reactions [61]. Hence, it was possible to exclude that the incomplete reaction of norbornene double bonds could be ascribable to disulfide bond formation, which might in turn lead to an impaired consumption of functional moieties. Conversely, the simultaneous presence of both unreacted thiol groups and norbornene double bonds further supported the hypothesis formulated on difficulties in the exposure of norbornene double bonds due to chain rigidity.

### 3.5. The Influence of Vis Light-Induced Photo-Crosslinking Mechanism Based on the -Ene Moiety

**Figure 12:**
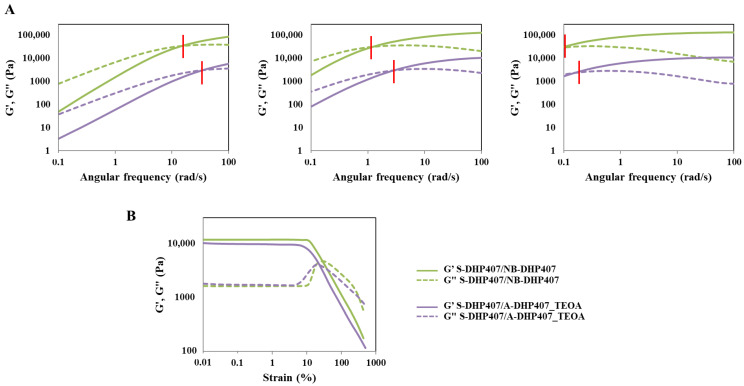
Frequency sweep tests at 25 °C, 30 °C and 37 °C (from left to right) (**A**), and strain sweep test at physiological temperature (**B**) performed on S-DHP407/NB-DHP407 (green) and S-DHP407/A-DHP407_TEOA (violet) formulations after exposure to visible light (525 nm, 80,000 Lux, 10 min). For higher clarity, G’ and G” values of S-DHP407/A-DHP407_TEOA formulations measured through frequency sweep test were divided by a factor of 10.

Differences in hydrogel mechanical properties ascribed to the kind of -ene moiety (i.e., acrylate- or norbornene-double bond) used for hydrogel design were studied through rheological tests performed on photo-irradiated S-DHP407/A-DHP407_TEOA and S-DHP407/NB-DHP407 formulations (Figure 12).

Irrespective of tested temperature, G’ and G” profiles acquired through frequency sweep tests showed lower ω_crossover_ values for thiol/norbornene systems compared to thiol/acrylate ones, thus suggesting the formation of hydrogels characterized by improved mechanical properties (Figure 12A). Strain sweep tests performed at physiological temperature further confirmed the formation of stronger S-DHP407/NB-DHP407 systems and the achievement of enhanced mechanical properties with strain at break measured to be 11.6% and 7.2% for thiol/norbornene and thiol/acrylate formulations, respectively (Figure 12B).

**Figure 13:**
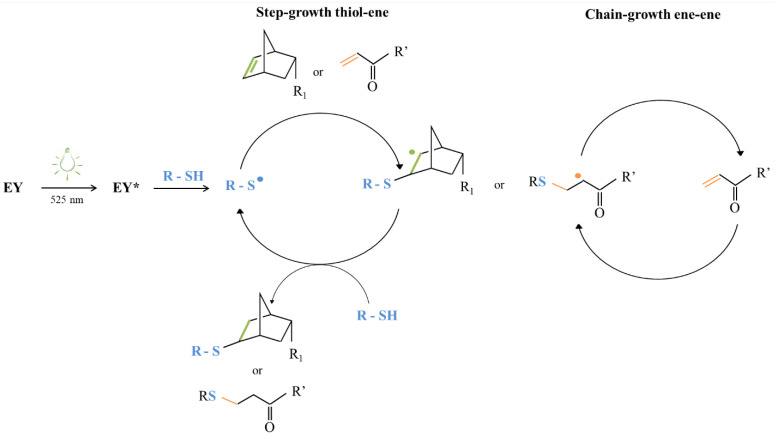
Schematic representation of the photo-crosslinking mechanism occurring based on the -ene moiety: purely step-growth photo-polymerization in the case of thiol-norbornene formulations and a mix mode, i.e., step-growth and chain-growth, in the case of thiol-acrylate systems.

As both thiol-ene formulations were based on a common PEU backbone functionalized to expose different photo-sensitive groups, differences in hydrogel mechanical properties could be mainly attributed to the photo-induced thiol-ene interaction mechanism (Figure 13). More in detail, light irradiation first makes the photo-initiator (i.e., EY) able to achieve an excited state (i.e., EY*), which in turn subtracts a hydrogen atom from thiol groups, thus leading to the formation of thiyl radicals (i.e., R-S^•^). Then, such reactive species are able to attack the norbornene or acrylate double bond forming carbon-centered radicals. At this step, different photo-polymerization routes occur based on the -ene moiety.

Indeed, as previously discussed, the interactions occurring between thiols and norbornene double bonds result in a purely step-growth polymerization [58]. On the other hand, the photo-induced crosslinking between thiols and acrylate double bonds leads to a mix-mode, i.e., the combination of step-growth and chain-growth mechanisms [63]. Specifically, the first reaction type (i.e., reaction between thiols and norbornene double bonds) led to the formation of bio-orthogonal networks, while the second one (i.e., reaction between thiols and acrylate double bonds) enhanced the formation of heterogeneities and defects, which resulted in worsened mechanical properties (i.e., lower critical deformation, achievement of a less developed gel structure). Hence, although the same thiol-ene photo-click chemistry was exploited to prepare the hydrogels, this study effectively demonstrated the capability to tune hydrogel final mechanical properties by selecting specific functional groups to react with.

## 4. Conclusions

In this work, dual stimuli-responsive formulations with promising features as mini-invasive delivery carriers or biomaterial inks/bioinks were successfully engineered by exploiting the versatility of poly(urethane) and water-based carbodiimide chemistries. Starting from an amphiphilic PEU bearing secondary amines along its backbone, three different photo-sensitive polymers were obtained through carbodiimide-mediated TGA, CEA and NBE coupling. Reactions were optimized to achieve successful PEU functionalization preserving group functionalities. Functionalized polymers were blended to develop two thiol-ene formulations undergoing gelation upon visible light irradiation, for exploitation as cell/biomolecule/drug carriers with no risks for photo-crosslinking induced damage/degradation. Specifically, the exposure to green light enhanced the formation of stronger gel networks with improved capability to withstand applied deformation compared to their corresponding not-irradiated systems. The addition of a co-initiator improved the photo-crosslinking mechanism of thiol-acrylate formulations, while less developed thiol-norbornene gels were obtained at 37 °C. Moreover, a fine tuning of hydrogel mechanical properties was successfully achieved based on the -ene moiety because of their different photo-crosslinking mechanism (i.e., step-growth and a combination of step- and chain-growth for thiol-norbornene and thiol-acrylate formulations, respectively). In conclusion, our results represent a promising milestone towards the ad-hoc design of multi-stimuli sensitive hydrogels with a double cross-linked network relying on both physical and chemical cross-links. The developed formulations will find application as injectable drug delivery systems or as biomaterial inks/bioinks in the 3D bioprinting of multi-layered structures for tissue engineering or regenerative pharmacology applications.

## Figures and Tables

**Table 1:** Acronyms adopted to refer to rheologically characterized thiol-ene PEU-based formulations and their corresponding composition.

	**Thiol-Acrylate formulations**
**S-DHP407/A-DHP407_ctrl**	S-DHP407 and A-DHP407 dissolved in PBS solution, mixed at 1:1 molar ratio (18% *w/v* overall concentration) and added with 1 mM EY
**S-DHP407/A-DHP407_light**	S-DHP407 and A-DHP407 dissolved in PBS solution, mixed at 1:1 molar ratio (18% *w/v* overall concentration), added with 1 mM EY and exposed to green light (525 nm) at 80,000 Lux for 10 min
**S-DHP407/A-DHP407_TEOA_ctrl**	S-DHP407 and A-DHP407 dissolved in PBS solution, mixed at 1:1 molar ratio (18% *w/v* overall concentration) and added with 1 mM EY and 7.5 mM TEOA
**S-DHP407/A-DHP407_TEOA_light**	S-DHP407 and A-DHP407 dissolved in PBS solution, mixed at 1:1 molar ratio (18% *w/v* overall concentration), added with 1 mM EY and 7.5 mM TEOA and exposed to green light (525 nm) at 80,000 Lux for 10 min
	**Thiol-Norbornene formulations**
**S-DHP407/NB-DHP407_ctrl**	S-DHP407 and NB-DHP407 dissolved in PBS solution, mixed at 1:1 molar ratio (18% *w/v* overall concentration) and added with 0.5 mM EY
**S-DHP407/NB-DHP407_light**	S-DHP407 and NB-DHP407 dissolved in PBS solution, mixed at 1:1 molar ratio (18% *w/v* overall concentration), added with 0.5 mM EY and exposed to green light (525 nm) at 80,000 Lux for 10 min
**S-DHP407/NB-DHP407_TYR_ctrl**	S-DHP407 and NB-DHP407 dissolved in PBS solution, mixed at 1:1 molar ratio (18% *w/v* overall concentration) and added with 0.5 mM EY and 0.1 mM TYR
**S-DHP407/NB-DHP407_TYR_light**	S-DHP407 and NB-DHP407 dissolved in PBS solution, mixed at 1:1 molar ratio (18% *w/v* overall concentration), added with 0.5 mM EY and 0.1 mM TYR and exposed to green light (525 nm) at 80,000 Lux for 10 min

**Table 2:** Crossover frequency between G’ and G’’ trends (ω_crossover_) measured for thiol-acrylate and thiol-norbornene formulations before and after exposure to visible light (525 nm, 80,000 Lux, 10 min) in the presence of a co-initiator (i.e., TEOA and TYR).

	ω_crossover_ (rad/s)		
	@ 25 °C	@ 30 °C	@ 37 °C
**S-DHP407/A-DHP407_TEOA_ctrl**	>100	7.2	0.36
**S-DHP407/A-DHP407_TEOA_light**	34	2.8	0.16
**S-DHP407/NB-DHP407_TYR_ctrl**	80	6.0	0.32
**S-DHP407/NB-DHP407_TYR_light**	38	2.8	0.17

## Data Availability

The data presented in this study are available on request from the corresponding authors.

## Supplementary Materials

The following supporting information can be downloaded at: https://www.mdpi.com/article/10.3390/ma16052024/s1, Figure S1: Evaluation of the thermo-responsiveness of functionalized poly(ether urethane)s at the nano-scale; Figure S2: Strain sweep test of thiol-acrylate photo-click hydrogels; Figure S3: Strain sweep test of thiol-norbornene photo-click hydrogels; Figure S4: Rheological assessment of the role exerted by the co-initiator in the thiol-ene photo-crosslinking process.
