# Supplementary material for: Thiol-Ene Photo-Click Hydrogels with Tunable Mechanical Properties Resulting from the Exposure of Different -Ene Moieties through a Green Chemistry
Source: Materials (Basel). 2023 Feb 28;16(5):2024. doi: 10.3390/ma16052024 (PMC10004734; doi:10.3390/ma16052024)
Supplement: Supplementary file 1 [file materials-16-02024-s001.zip › materials-2221604-supplementary.pdf]

Thiol-Ene Photo-Click Hydrogels with Tunable Mechanical Properties Resulting from the Exposure of Different -Ene Moieties through a Green Chemistry

Rossella Laurano [1,†,*], Monica Boffito [1,†], Claudio Cassino [2], Ludovica Midei [1], Roberta Pappalardo [1,3], Valeria Chiono [1] and Gianluca Ciardelli [1,*]

[1] Department of Mechanical and Aerospace Engineering, Politecnico di Torino, 10129 Turin, Italy
[2] Department of Science and Technological Innovation, Università del Piemonte Orientale, 15121 Alessandria, Italy
[3] Department of Surgical Sciences, Università degli Studi di Torino, 10126 Turin, Italy
* Correspondence: rossella.laurano@polito.it (R.L.); gianluca.ciardelli@polito.it (G.C.)
† These authors equally contributed to this work

Evaluation of the thermo-responsiveness of functionalized poly(ether urethane)s at the nano-scale

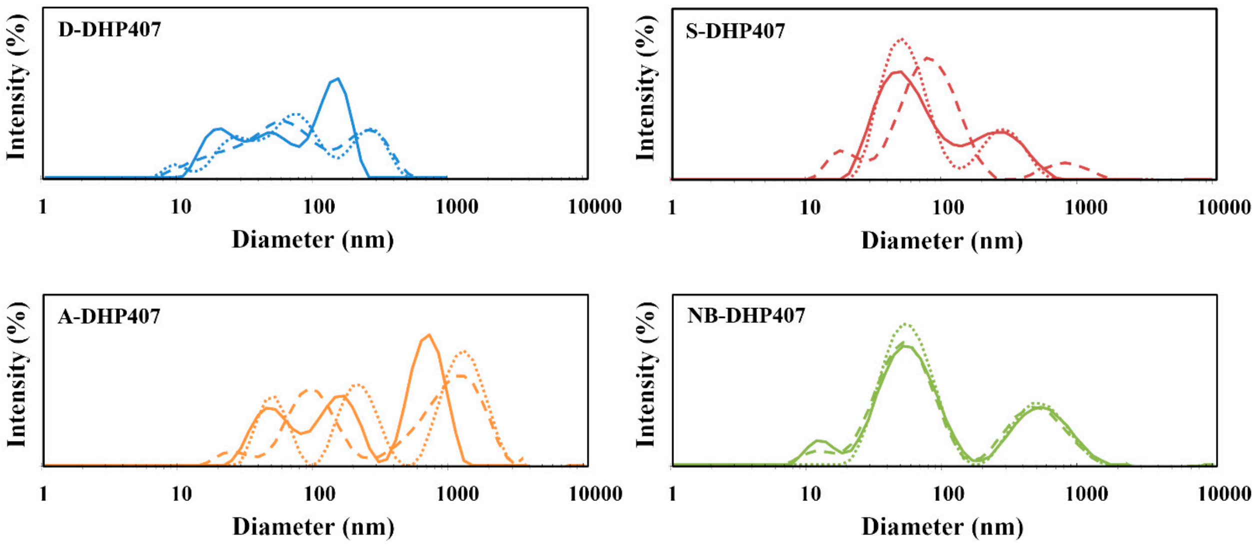

Figure S1. Intensity patterns of the polymeric structures contained in D-DHP407 (blue), S-DHP407 (red), A-DHP407 (orange) and NB-DHP407 (green) solutions (0.5% w/v polymeric concentration) as measured through DLS analysis at 25 °C. In each graph, continuous, dotted and dashed lines represent profiles acquired by analyzing three independent samples.

Thiol-acrylate photo-click hydrogels

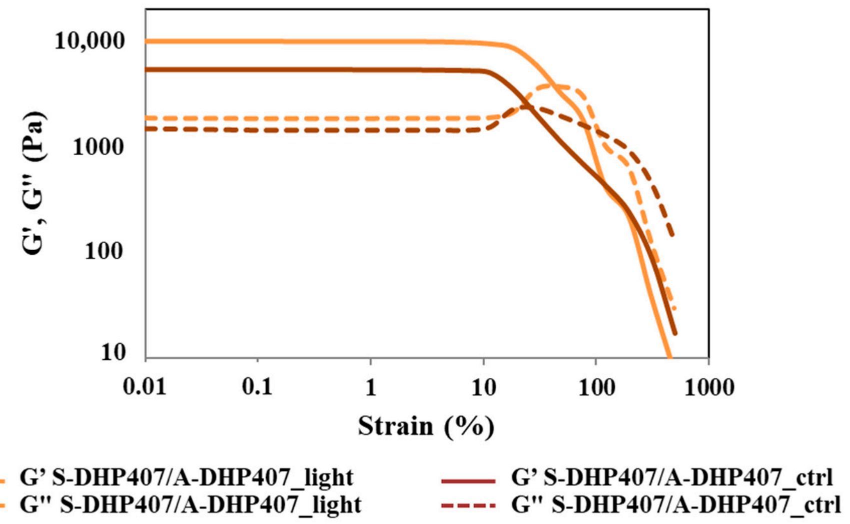

Figure S2. Strain sweep test at 37 °C performed on S-DHP407/A-DHP407 before (dark orange) and after (light orange) exposure to green light (525 nm, 80000 Lux, 10 min). Continuous and dashed lines represent the storage (G') and loss (G") moduli, respectively.

Thiol-norbornene photo-click hydrogels

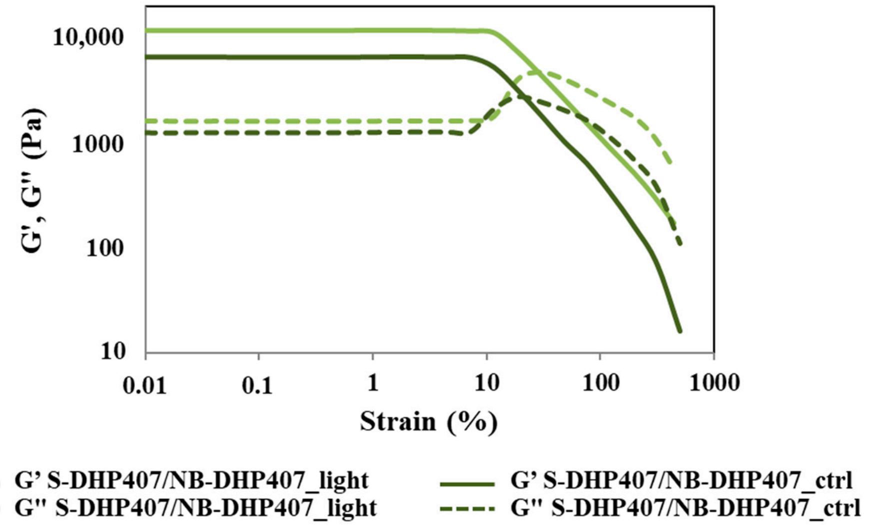

Figure S3. Strain sweep test performed at 37 °C on S-DHP407/NB-DHP407 before (dark green) and after (light green) exposure to green light (525 nm, 80000 Lux, 10 min). Continuous and dashed lines represent the storage (G') and loss (G") moduli, respectively.

The role exerted by the co-initiator in the thiol-ene photo-crosslinking process

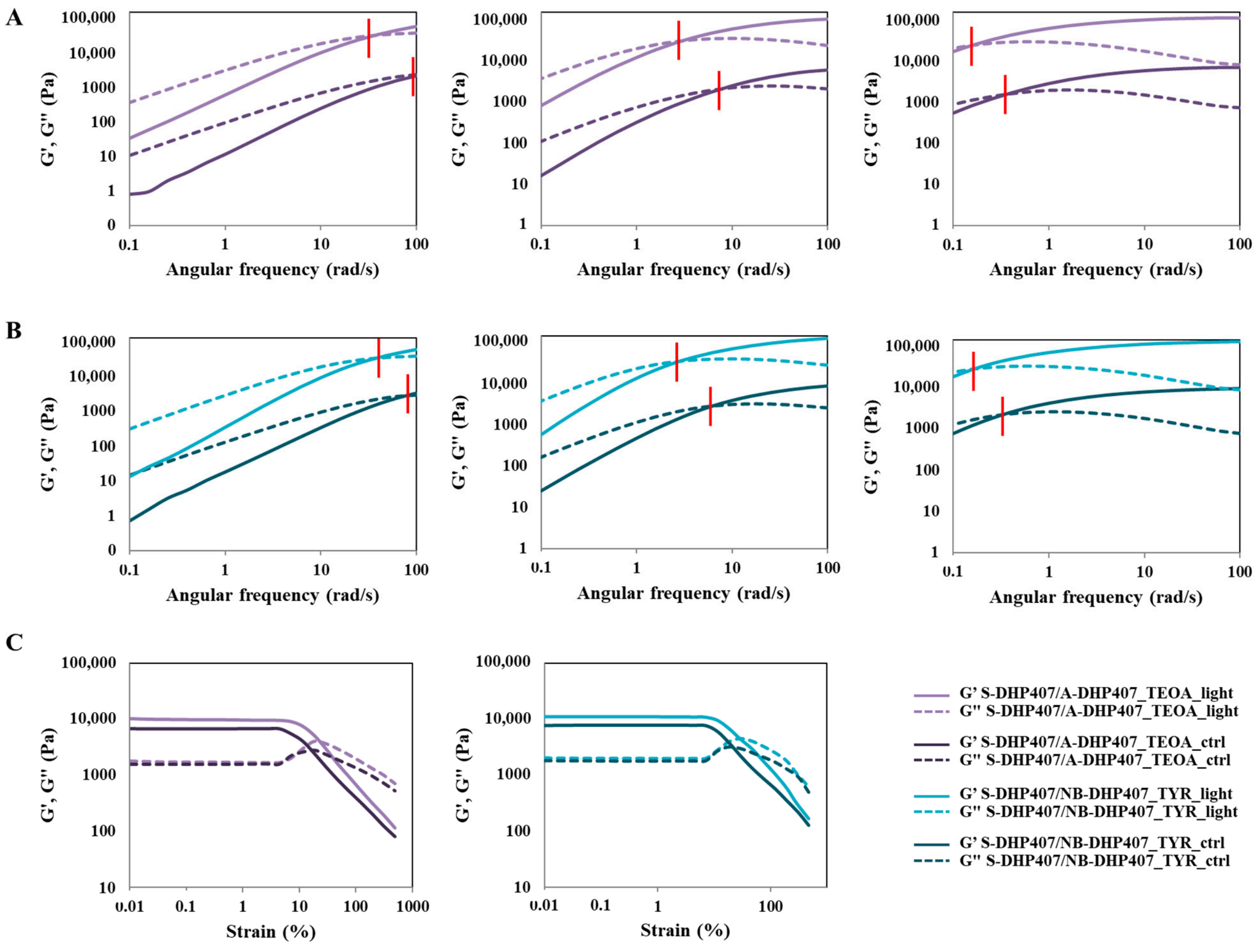

Figure S4. Frequency sweep tests performed at 25 °C, 30 °C and 37 °C (from left to right) on S-DHP407/A-DHP407 (A) and S-DHP407/NB-DHP407 (B) formulations before (dark colors) and after (light colors) exposure to visible light (525 nm, 80000 Lux, 10 min) upon the addition of a co-initiator (i.e., TEOA and TYR for thiol-acrylate and thiol-norbornene systems, respectively). For higher clarity, G' and G" values of control samples were divided by a factor of 100. Irrespective of considered formulation and temperature, the addition of a co-initiator did not alter formulation capability to undergo a temperature-driven gelation both pre- and post-irradiation. Moreover, the comparison between each light-irradiated formulation and its corresponding control suggested the successful thiol-ene interaction through the $\omega_{crossover}$ shift towards lower frequencies. (C) Strain sweep test performed at 37 °C on S-DHP407/A-DHP407_TEOA (left) and S-DHP407/NB-DHP407_TYR (right) formulations before (dark colors) and after (light colors) light-irradiation.
